# Revealing acute consequences of rapid degradation of synaptic fusion proteins at individual synapses using Auxin-Inducible Degron 2 technology

**DOI:** 10.1038/s42003-025-08996-8

**Published:** 2025-11-17

**Authors:** Lilach Elbaum-Mendelson, Weixiang Yuan, Johannes P.-H. Seiler, Nadia Blom, Ya-Chien Chan, Ali Hyder Baig, Nils Brose, Simon Rumpel, Noam E. Ziv

**Affiliations:** 1 Technion Rappaport Faculty of Medicine and Network Biology Research Laboratories, Fishbach Building, Technion City, Haifa, Israel; 2 Institute of Physiology, Focus Program Translational Neurosciences, University Medical Center of the Johannes Gutenberg University Mainz, Mainz, Germany; 3 Department of Molecular Neurobiology, Max Planck Institute for Multidisciplinary Sciences, Göttingen, Germany

**Keywords:** Cellular neuroscience, Microscopy

## Abstract

Roles of particular proteins in synaptic organization and function are commonly studied by knock-out, knock-down or overexpression strategies. Such approaches are typically protracted, associated with adaptive changes and challenge the ability to observe acute consequences at individual synapses. Here we describe the use of Auxin-Inducible Degron 2 (AID2) technology and coexpressed reporters to study real-time effects of rapidly degrading postsynaptic density proteins at individual synapses. We establish the capacity of AID2 technology to rapidly degrade postsynaptic scaffold fusion proteins in cultured neurons and in vivo. We show that acute PSD-95 or gephyrin degradation leads to concomitant loss of AMPA or GABA_A_ receptors from the same synapses. Unexpectedly, we find that acute GKAP, but not PSD-95 degradation, is associated with scaffold size reductions at the same synapses. Our findings demonstrate the utility of approaches based on acute degradation and live imaging for studying the roles of select proteins in synaptic organization.

## Abstract (web summary)

Roles of specific synaptic proteins in postsynaptic organization revealed by acute, Auxin-Inducible Degron 2 Technology-mediated degradation of synaptic fusion proteins, combined with real time measurements of consequences at individual synapses.

## Introduction

Mammalian synapses are minute cellular specializations composed of hundreds of protein species that play myriad, often overlapping roles in neurotransmitter secretion and reception, functional regulation and in maintaining synaptic organization in face of incessant protein dynamism and turnover^1^. Among the most powerful approaches for elucidating the importance and functions of such proteins are subtractive (eliminative) and additive approaches, that is, examining how the loss or excess of a protein of interest (POI) affect the organization, function and stability of synaptic multimolecular complexes, organelles and synapses.

Where subtractive approaches are concerned, constitutive or conditional knock-out approaches that act at the genome level are considered the gold standard. These approaches, however suffer from several inherent drawbacks, the main being the protracted time course of POI elimination. Put differently, the time interval between the manipulation and its manifestation or assessment is typically long, due to the time course of organism development, in the case of constitutive knock-outs, or the long lifetimes and consequential protracted degradation time courses of most synaptic proteins^2^ in the case of conditional knock-outs. These protracted time courses leave ample time for slow compensatory and adaptive processes that often obfuscate the direct consequences of POI loss to synaptic organization, function and properties and thus complicate interpretations of such experiments. Other drawbacks include the complexity of genomic level manipulations and their typically irreversible nature. Consequently, alternative technologies were developed for eliminating mRNAs encoding for the POI (RNA interference, or ‘knock-down’^3^). But here again, POI loss is typically drawn-out for the same reasons, that is, dependence on slow, endogenous clearance pathways. Moreover, off-target effects are not uncommon^4^. Finally, from an experimental viewpoint, the aforementioned protracted time scales severely challenge the ability to record the consequences of POI loss to the properties of individual synapses, cells or networks throughout the POI removal process.

A different set of subtractive approaches aim to target the POI itself through degradation or inactivation. Such approaches typically involve the expression of fusion proteins of POIs with highly specific degrons or cleavage sites, which enable experimentally timed, rapid degradation or inactivation of the fusion proteins. Although the utility of this approach has long been recognized^5,6^, its application to synaptic biology has been rare^5–9^, and almost never included measurements made at the same synapses.

Where additive approaches are concerned, a common approach is to overexpress a synaptic POI and examine how this affects synaptic organization and function. In common with knockout and knockdown approaches, overexpression kinetics are protracted and often associated with slow reorganization processes, during which synapses, neurons and networks react to POI excess and gradually settle at new steady states. Importantly, these steady states are dictated by the properties of the POI and those of proteins it interacts with, and are thus informative. The reactive processes and their protracted time scales, however, complicate interpretations and the ability to separate direct effects from indirect consequences of POI overexpression.

A radically different additive approach for studying the roles of specific POIs in synaptic organization involves in-vitro, biochemical reconstitution of synaptic multimolecular complexes using mixtures of purified proteins or protein fragments, often fused to fluorescent proteins. This approach, most recently carried out within a framework positing that many synaptic multimolecular complexes (both pre and postsynaptic) are effectively condensates formed through liquid-liquid phase separation (LLPS), has recently proved to be enormously informative^10–20^ (reviewed in refs. ^21–24^). Furthermore, by omitting particular proteins from such mixtures, clues as to which synaptic proteins might act as ‘drivers’ of synaptic organization (i.e. induce condensate formation) or join as relatively passive ‘clients’ have been obtained^10^. While this approach is highly informative, these biochemical assays are typically performed using truncated proteins or fused protein fragments within greatly simplified environments, devoid of hundreds of other synaptic molecules and the complex intracellular settings of intact synapses and neurons.

A potentially useful synthesis of the aforementioned approaches would be to 1) overexpress, in living neurons, synaptic proteins at combinations guided by biochemical reconstitution experiments, among others, allowing ample time for the overexpressed POIs to impose new steady states that accentuate their roles and interactions with other synaptic proteins; 2) use targeted synaptic protein degradation to rapidly degrade an overexpressed POI; and 3) follow the acute consequences in situ - at the same synapses and cells, using coexpressed synaptic proteins as real-time reporters.

Here we demonstrate the utility of this approach in combination with Auxin-Inducible Degron 2 (AID2) technology^25^ for studying the roles of specific postsynaptic scaffold proteins in synaptic organization and their capacity to act as drivers in this regard - a topic explored historically using knockout (e.g. refs. ^26–29^), knockdown (e.g. refs. ^26,30–33^) and overexpression (e.g. refs. ^34–41^) approaches, among others. Given recent biochemical reconstitution studies pointing to the unique capacities of the postsynaptic density (PSD) proteins GKAP^10,13^ and gephyrin^20,42^ to serve as drivers of postsynaptic organization, we expressed, in cortical neurons, combinations of PSD-95, GKAP (excitatory synapses), and Gephyrin (inhibitory synapses) as well as subunits of their cognate neurotransmitter receptors (GluA2 and GABA_A_Rα_2_, respectively) fused to fluorescent reporters. After allowing ample time for steady states to be reached, we drove the rapid degradation of each of the PSD fusion molecules using AID2 technology, and measured the acute, real-time consequences at individual synapses. We first demonstrate the robust capacity of AID2 technology to acutely and reversibly degrade postsynaptic scaffold proteins fused to GFP variants or HaloTags in cultured neurons and in vivo. We then show that rapid degradation of PSD-95 and Gephyrin fusion proteins results in the loss of glutamate and GABA receptors, respectively, from the same postsynaptic sites, even in the presence of endogenous variants of the same proteins. We then explore the degree to which the rapid degradation of PSD-95 or GKAP fusion proteins affects PSD size, finding, that the acute loss of PSD-95 does not appear to reduce PSD size and, in fact, is associated with an influx of other PSD molecules into the same postsynaptic sites. Conversely, rapid degradation of GKAP causes significant PSD shrinkage and is associated with PSD-95 efflux from the same postsynaptic sites. Our findings highlight the reliability and robustness of the AID2 system for targeting synaptic POIs, and demonstrate the utility of approaches based on acute POI degradation and live imaging for studying the roles of select proteins in synaptic organization.

## Results

### PSD proteins are degraded rapidly by the AID2 system

AID2 technology^25^ is based on three components taken from a ubiquitous signaling cascade in plants, induced by plant Auxin hormones: (i) the *Arabidopsis*-derived mini-AID (mAID) degron, which is fused to the POI, (ii) a modified E3 ubiquitin ligase from rice (*Oryza sativa* transport inhibitor response 1, or OsTIR1), which forms a Skp1-Cul1-F-box (SCF) E3 ligase complex with endogenous components, and (iii) the Auxin derivative 5-phenyl-indole-3-acetic acid (5-Ph-IAA). In the presence of 5-Ph-IAA, the E3 complex binds to the mAID degron and ubiquitinates it, resulting in proteasomal degradation of the fusion protein. AID2 is a second-generation AID technology^43^, based on mutation of OsTIR1 (F74G) and modification of its ligand, which resolved prior problems such as leaky degradation and dependence on high inducer concentrations. Although potentially powerful, the use of AID2 technology in the field of neuroscience has been sparse^25,44,45^, and to the best of our knowledge, has not been used yet to study synapses, possibly due to the leakiness exhibited by the first-generation system^6^.

We first validated published mAID2 tools that had previously been tested in cultured mouse hippocampal neurons^25^. We obtained the original pAAV-hSyn-OsTIR1(F74G) plasmid from a public repository (see Methods), and moved the coding region from an AAV to a lentiviral backbone. This vector codes for OsTIR1(F74G) and, separated by a P2A sequence, EGFP fused on its N-terminal to the mAID degron and on it C-terminus to a nuclear export signal (OsTIR1-P2A-mAID:EGFP:NES; Supplementary Fig. 1A). When this construct is expressed in cultured rat cortical neurons, exposure to 200 nM 5-Ph-IAA is followed by the rapid (<2 h) disappearance of EGFP, in agreement with the original report^25^ (two separate experiments, 13 and 17 neurons, control and 5-Ph-IAA-treated, respectively; Supplementary Fig. 1B, C).

**Fig. 1:**
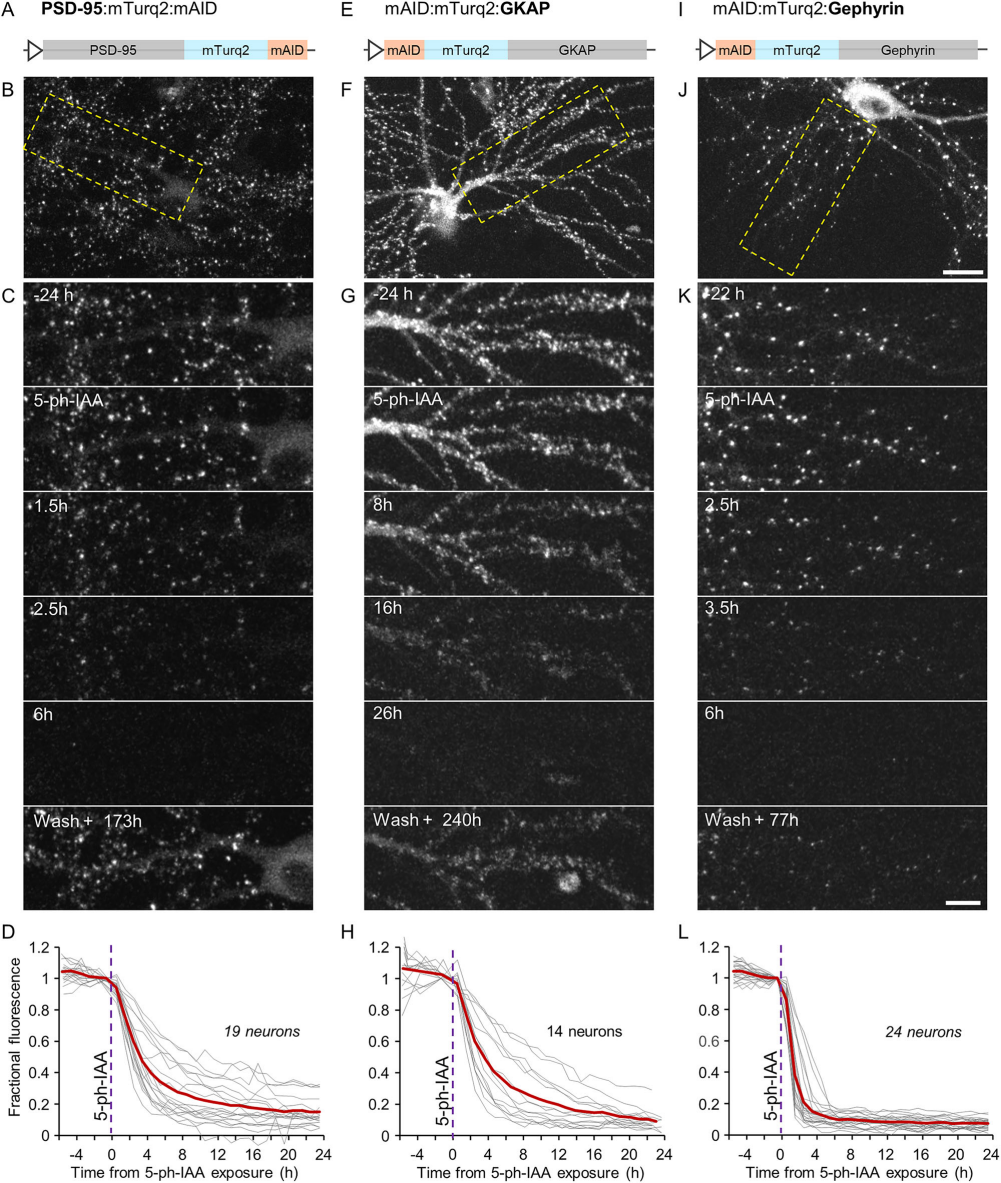
Rapid degradation of postsynaptic scaffold proteins fused to mTurquoise2 and a mAID degron. **A** Illustration of the PSD:95, mTurquoise2 and mAID fusion protein (PSD-95:mTurq2:mAID). **B** A rat cortical neuron in culture expressing PSD-95:mTurq2:mAID as well as OsTIR1-P2A-mAID:EGFP:NES (see Supplementary Fig. 1A). **C** Region in yellow rectangle in (**B**) at greater detail, before, after addition of 5-Ph-IAA, and after washing out the 5-Ph-IAA. Note the nearly complete loss of PSD-95:mTurq2:mAID fluorescence, and its recovery after several days. **D** PSD-95:mTurq2:mAID fluorescence measured at 30 synapses of each neuron tracked throughout the experiments. Each thin gray line is the average fluorescence measured for the synapses of one neuron. Fluorescence values for each neuron were normalized to the fluorescence measured at the last time point before 5-Ph-IAA was added. Thick red line - population average (19 neurons from 3 independent experiments, 570 synapses in total). **E**–**H** As in A-D for mAID:mTurq2:GKAP. 14 neurons from 2 independent experiments, 10–19 synapses per neuron, 199 in total). **I–L** As in (**A**–**D**) for mAID:mTurq2:Gephyrin. 24 neurons from 3 independent experiments, 30 synapses per neuron, 720 synapses in total. Scale bars: 20 µm (**B**, **F**, **J**) and 10 µm (**C**, **G**, **K**).

To test the functionality of the AID2 system for degradation of synaptic proteins, we generated a lentiviral vector encoding a fusion protein of PSD-95, the fluorescent protein mTurquoise2^46^, and a C-terminus mAID tag (PSD-95:mTurq2:mAID; Fig. 1A). PSD-95 is a prototypical PSD scaffold protein of glutamatergic synapses, which plays a crucial role in organizing synaptic components, in particular the confinement of multiple classes of glutamate receptors to postsynaptic membranes^31,32,47–49^ (reviewed in refs. ^50,51^). When expressed in cultured rat cortical neurons, PSD-95:mTurq2:mAID localized to postsynaptic sites along dendrites as previously observed for a PSD-95:mTurq2 variant lacking the mAID degron^52^. To follow the 5-Ph-IAA induced degradation of PSD-95:mTurq2:mAID and the corresponding kinetics, cortical neurons were cotransduced with lentiviruses coding for PSD-95:mTurq2:mAID and lentiviruses coding for OsTIR1-P2A-mAID:EGFP:NES, to introduce the essential OsTIR1(F74G) ubiquitin ligase and serve as positive controls. The preparations were mounted on a confocal microscope, connected to a slow perfusion system, and maintained at ~37 °C in an atmospheric environment of 5% CO_2_. After collecting baseline images at 1–2 h intervals for a few hours, 5-Ph-IAA (200 nM) was added to the preparations and the neurons were imaged at 1 h intervals. As shown for one neuron in Fig. 1B, C, and for individually followed synapses (*n* = 570, 19 neurons, 3 independent experiments; Fig. 1D), exposure to 5-Ph-IAA was followed by the nearly complete degradation of PSD-95:mTurq2:mAID over 8–16 h, with PSD-95:mTurq2:mAID fluorescence exhibiting an approximately exponential decay curve with a time constant of ~3.7 h (see Methods).

A second excitatory synapse PSD protein we tested here is GKAP (also known as SAPAP1, DLGAP1 and DAP1). GKAP^53^ is thought to bridge a membrane-proximal layer of scaffold proteins, including glutamate receptors and PSD-95, and a membrane distal layer, including SHANKs/ProSAPS and cytoskeletal linkers^54^. Importantly, GKAP was reported to be a key organizer of the glutamatergic synapse PSD^10,13,33,55^. As a previous study showed that GKAPs function is maintained following N-terminal tagging^56^, we created a N-terminus fusion protein of mAID, mTurquoise2 and GKAP (mAID:mTurq2:GKAP), which also provided an opportunity to test the utility of N-terminal mAID tags for AID2-based synaptic POI degradation. When mAID:mTurq2:GKAP was expressed in cortical neurons (together with OsTIR1-P2A-mAID:EGFP:NES), mAID:mTurq2:GKAP assumed a postsynaptic localization (Fig. 1F, G), as previously seen with EGFP- and YFP-tagged GKAP^56,57^. The addition of 5-Ph-IAA led to the rapid degradation of mAID:mTurq2:GKAP (Fig. 1E–H). When mAID:mTurq2:GKAP fluorescence was followed at individual synapses, it was observed to follow an approximately exponential decay with a time constant similar to that observed for PSD-95:mTurq2:mAID (~3.7 h; 199 synapses from 14 neurons from two independent experiments).

PSD-95 and GKAP are well established components of excitatory glutamatergic synapses. Analogously, Gephyrin is considered to be the main organizer of the postsynaptic specialization at inhibitory GABAergic and glycinergic synapses, confining ionotropic GABA and Glycine receptors to postsynaptic sites^42,58–62^. To examine if AID2 technology can be used to target Gephyrin as well, we tested a fusion protein of mAID, mTurquoise2 and Gephyrin, with the mAID and mTurquoise2 fused to the N-terminus of Gephyrin (mAID:mTurq2:Gephyrin; Fig. 1I), in accordance with prior studies^63,64^. When expressed in cortical neurons, mAID:mTurq2:Gephyrin assumed a punctate, dendritic expression pattern (Fig. 1J), identical to that observed for the same fusion protein lacking mAID^64^. Again, in neurons coexpressing OsTIR1-P2A-mAID:EGFP:NES, 5-Ph-IAA induced very rapid degradation of mAID:mTurq2:Gephyrin (Fig. 1I–L) with an approximate time constant of 1.6 h (720 synapses from 24 neurons from 3 separate experiments).

**Fig. 2:**
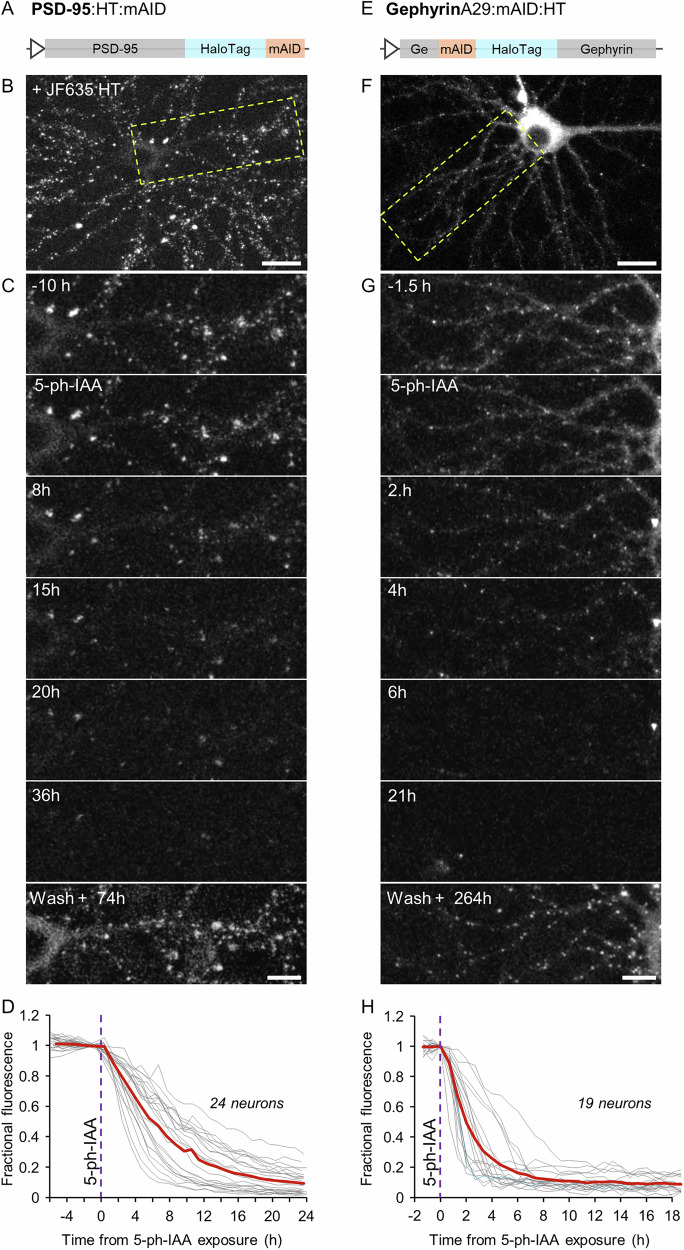
Rapid degradation of postsynaptic scaffold proteins fused to HaloTag protein and a mAID degron. **A** Illustration of the PSD-95 - HaloTag - mAID fusion protein (PSD-95:HT:mAID). **B** A rat cortical neuron in culture expressing PSD-95:HT:mAID as well as OsTIR1-P2A-mAID:EGFP:NES. PSD-95:HT:mAID was rendered visible by labeling with JF635-HT (100 nM). **C** Region in yellow rectangle in (**B**) at greater detail, before, after addition of 5-Ph-IAA, and after washing out 5-Ph-IAA. Note the nearly complete loss of JF635-HT fluorescence, and its recovery after several days (following a second labeling with JF635-HT). **D** JF635-HT fluorescence measured at 11–30 synapses of each neuron (443 in total) tracked throughout the experiments. Each thin gray line is the average JF635-HT fluorescence measured for the synapses of one neuron. Fluorescence values for each neuron were normalized to the fluorescence measured at the last time point before 5-Ph-IAA was added. Thick red line is the population average (24 neurons from 3 independent experiments). **E–H** As in (**A**–**D**) for GephyrinA29:mAID:HT. 18 neurons from 3 independent experiments. Scale bars: 20 µm (**B**, **F**) and 10 µm (**C**, **G**).

Given the expanding use of HaloTag (HT) Technology^65^ to visualize POIs, we examined whether mAID2 technology can also be used to rapidly degrade HT-labeled synaptic scaffold POIs, particularly when the HT is bound to fluorescent HT ligands, which could potentially interfere with proteasomal degradation. To that end we substituted mTurq2 with HT in PSD-95:mTurq2:mAID, obtaining PSD-95:HT:mAID (Fig. 2A). In this case, neurons expressing PSD-95:HT:mAID together with OsTIR1-P2A-mAID:EGFP:NES were first exposed to the fluorescent ligand JF635-HT^66^, which led to the highlighting of postsynaptic sites (Fig. 2B). Exposure to 5-Ph-IAA led to the striking disappearance of ligand fluorescence, indicating that the fusion protein was degraded. The time constant of the fluorescence loss (~8.5 h; Fig. 2D), was somewhat longer than that observed for PSD-95:mTurq2:mAID (443 synapses from 24 neurons from 3 separate experiments).

Similarly, we generated a fusion protein of Gephyrin and a HaloTag, but decided to insert the mAID:HT sequence at a different site, after observing examples in which an antibody against gephyrin failed to recognize the N-terminal fusion protein variants described above (Supplementary Fig. 2A). We therefore inserted the mAID:HT sequence between Asparagine 29 and Leucine 30 (GephyrinA29:mAID:HT; Fig. 2E), following the strategy used to create an mRFP-tagged Gephyrin knock-in mouse^67^. As shown in Fig. 2F–H, GephyrinA29:mAID:HT assumed a dendritic, punctate expression pattern, similar to that observed with the original fusion proteins. Moreover, GephyrinA29:mAID:HT was now recognized by the anti-gephyrin antibody mentioned above (Supplementary Fig. 2B). Exposure of neurons co-expressing GephyrinA29:mAID:HT and OsTIR1-P2A-mAID:EGFP:NES caused the loss of ligand fluorescence, indicative of rapid degradation of the Gephyrin fusion protein (530 synapses from 19 neurons from 3 separate experiments). Here too, the kinetics of fluorescence loss were somewhat slower that those observed for gephyrin fused to mTurq2 (time constant of ~2.5 h).

One major advantage of AID2 technology over most knockout and knockdown approaches is the inherent reversibility of elimination it allows, realized simply by removing 5-Ph-IAA^45^. Indeed, when 5-Ph-IAA was washed out at the end of experiments, and the preparations were examined again after several days, clear recovery was observed for all the fusion proteins tested (Figs. 1C, G, K, and 2C, G; Note that JF635-HT was re-added to the dish to visualize the HT fusion proteins). The time course of recovery was not followed systematically due to the slow turnover rates of synaptic proteins, and was sampled at single time points. Recovery, however, was very robust for all fusion proteins examined.

Analyses of spatial protein degradation patterns for all fusion proteins did not reveal notable spatial gradients beyond a tendency of somatic, diffuse fluorescence loss to slightly precede fluorescence loss at synapses, and only minor, if any differences between fluorescence loss from proximal and distal synaptic sites. The spatial uniformity, as well as the abruptness and completeness of fluorescence loss, are demonstrated qualitatively in Supplementary Movie 1 and quantified in Supplementary Fig. 3.

In summary, mAID fusion proteins of excitatory and inhibitory synapse PSD POIs, labeled with either GFP variants or HT, and fused N-terminally, C-terminally, or intramolecularly, are rapidly and reversibly degraded in the presence of OsTIR1(F74G) and 5-Ph-IAA.

### Degradation of mAID fusion proteins in-vivo

**Fig. 3:**
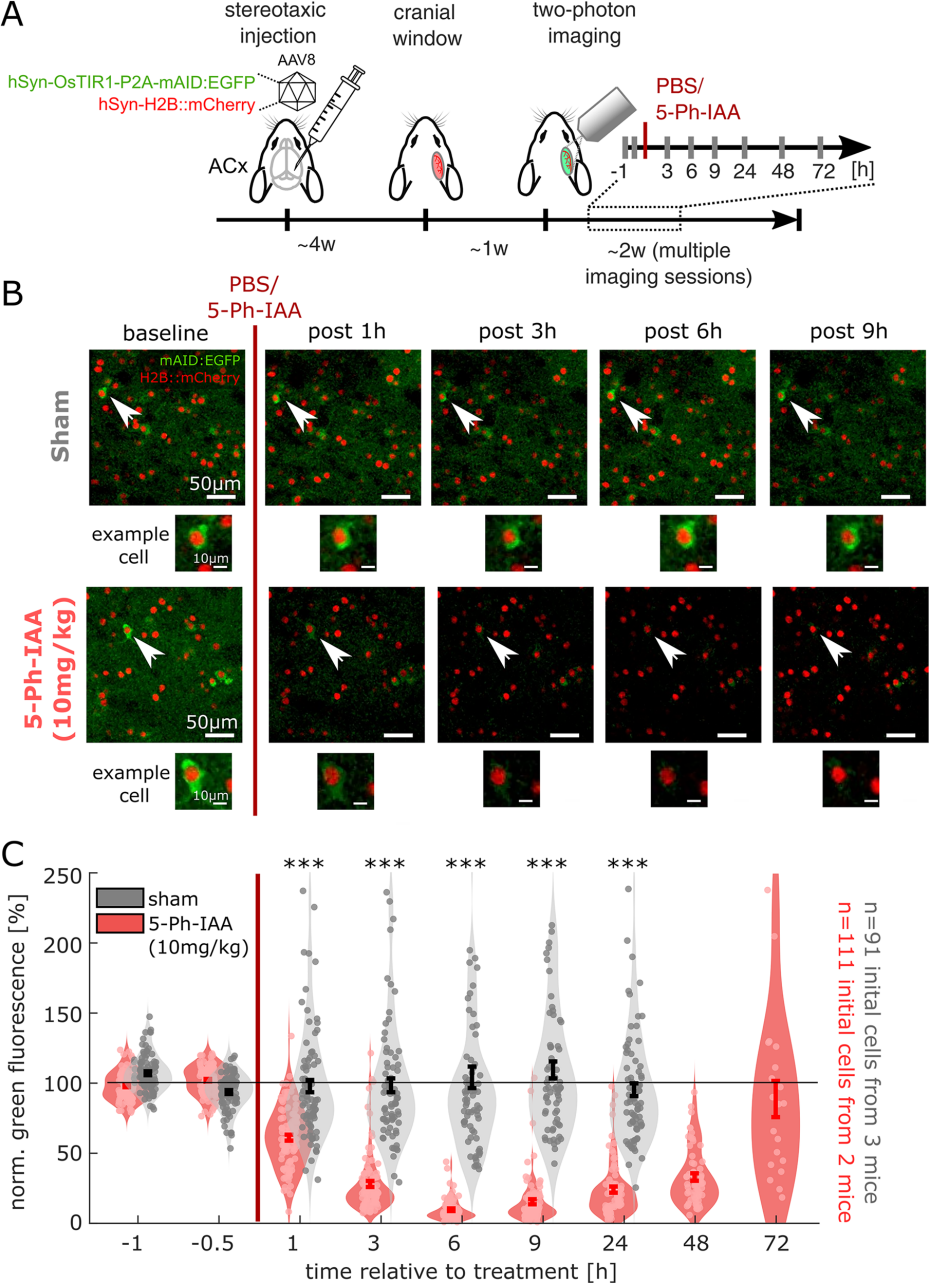
AID2-mediated degradation and recovery of a soluble protein in vivo. **A** Flow of experiment. C57BL/6J mice (*n* = 3) were injected with AAV vectors to cytosolically express mAID:GFP, OsTIR1 and a stable nuclear reference marker (H2B:mCherry) in the auditory cortex. Animals underwent cranial window implantation and longitudinal two-photon imaging after sham (PBS) or 5-Ph-IAA treatment. **B** Exemplary fields of view, showing stable mAID:EGFP fluorescence after sham treatment and vastly reduced mAID:EGFP fluorescence after auxin treatment. **C** Quantification of the normalized cytosolic mAID:EGFP fluorescence (see Methods) over the course of imaging (*n* = 91 sham neurons from 3 mice vs. 111 5-Ph-IAA treated neurons from 2 mice), showing a swift reduction of fluorescence over the course of few hours and a subsequent recovery after approximately three days (***p < 0.001, Wilcoxon rank sum test of sham vs. 5-Ph-IAA).

In their original study^25^, Yesbolatova and colleagues demonstrated the utility of the AID2 system to drive mAID-EGFP degradation in various organs of transgenic mice following intraperitoneal 5-Ph-IAA injection. mAID-EGFP degradation was also observed in whole brains, albeit to a somewhat lesser extent (~50%) than that observed in other organs (see also refs. ^45,68^). To examine the in-vivo efficacy of this system at the single neuron level and over time, we first packaged the OsTIR1-P2A-EGFP:mAID vector from the original study into an AAV capsid (see Methods) and then stereotaxically injected it into the auditory cortex of wildtype mice together with a second AAV vector, coding for Histone 2B fused to mCherry (H2B:mCherry). This combination led to the expression of a cytosolically expressed mAID:EGFP, together with a stably expressed nuclear reference marker (Fig. 3A, B). After a recovery period, we implanted the injected mice with a cranial window and conducted longitudinal two-photon imaging, which allowed a detailed assessment of the time course of the degradation and recovery over time in the same neurons. Specifically, we imaged immediately before (−1, −0.5 h) and repeatedly after (1, 3, 6, 9, 24, 48 and 72 h) intraperitoneal administration of 10 mg/kg 5-Ph-IAA (5-Ph-IAA group; 3 mice) or saline (sham group; 2 mice). In line with the original study reporting a decrease of GFP fluorescence by ~50% after sacrificing mice 6 h after injection^25^, we observed a substantial decrease of cytosolic mAID:EGFP fluorescence after injection of 5-Ph-IAA, but not with saline (Fig. 3B; 91 and 111 neurons, respectively). In our experiments, the loss of mAID:EGFP fluorescence in cell bodies and the neuropil became apparent already within one hour and reached a maximal reduction of ~90% after 6 h (*p* < 0.001; *t *= 1, 3, 6, 24 h). Moreover, following this initial reduction, a recovery of fluorescence was observed over the course of about three days (Fig. 3C). These observations demonstrate the efficient yet reversable depletion of a cytosolic protein using the AID2 system in individual neurons in-vivo.

**Fig. 4:**
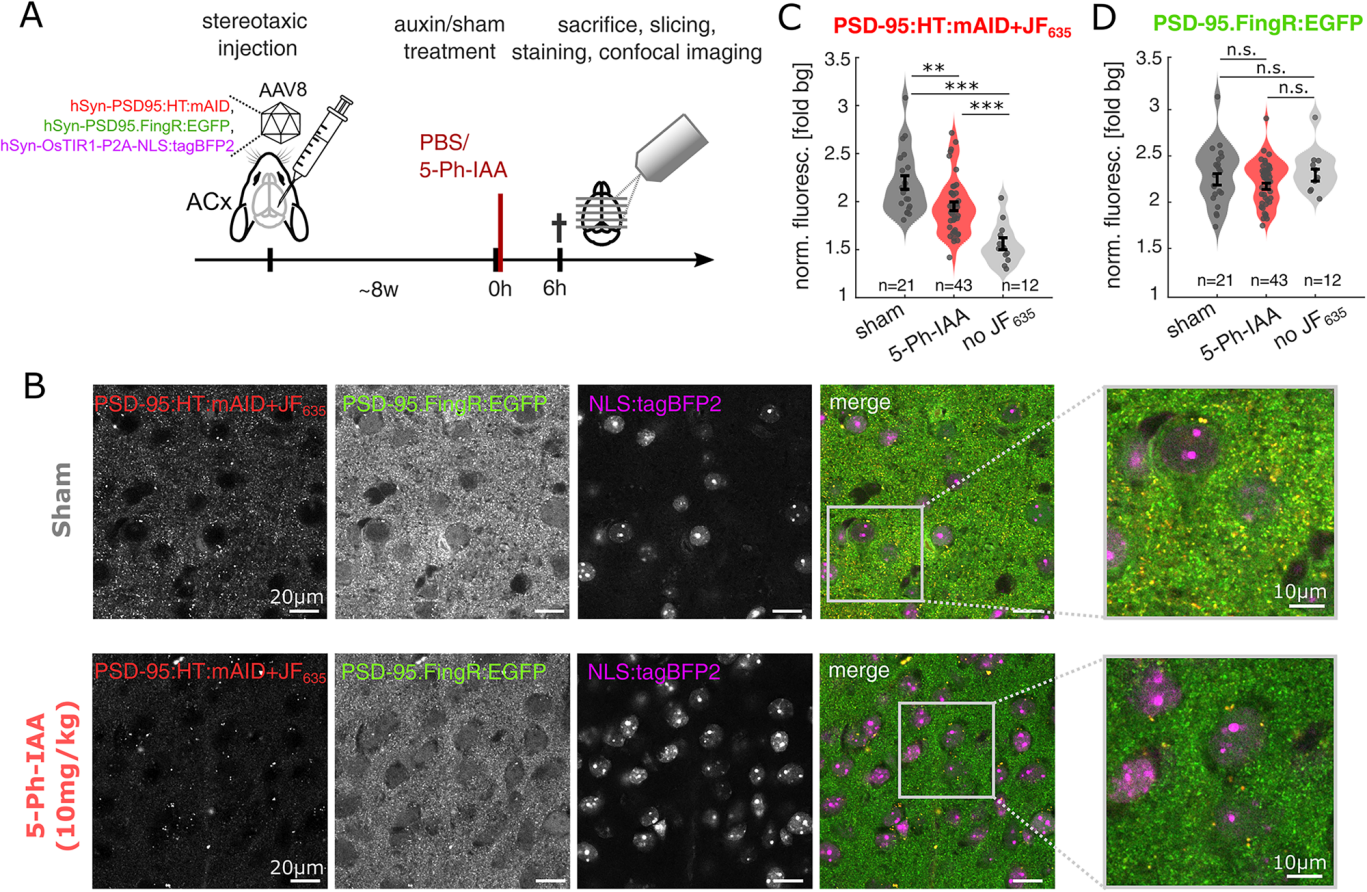
AID2-mediated degradation and recovery of a synaptic scaffold fusion protein in vivo. **A** Flow of experiment. After stereotaxic injection and stable expression of the exogenous proteins, mice were treated with (sham) or 5-Ph-IAA (auxin). After 6 h, mice were sacrificed and their brains were extracted, fixed and stained with the HaloTag ligand JF635-HT before starting confocal imaging (*n* = 3 sham mice vs. 4, 5-Ph-IAA mice). **B** Exemplary fields of view, showing all three imaging channels in a sham and 5-Ph-IAA treated animal. Right: Magnified image, showing the punctate pattern of PSD-95 signals, expected to correspond to excitatory synapses. The PSD-95:HT:mAID signal is decreased after the 5-Ph-IAA treatment compared to the sham treatment. **C** Quantification of the normalized PSD-95:HT:mAID fluorescence in the neuropil, showing a significant signal depletion after 5-Ph-IAA injection (n indicates the number of FOV per condition; **p < 0.01, ***p < 0.001 in Wilcoxon rank sum test). Note that degradation of PSD-95:HT:mAID may not have reached its maximum at this six hours observation period (see Fig. 2D). **D** Equivalent quantification for the PSD-95:FingR:EGFP signal, showing a slight, but not statistically-significant drop in intensity after 5-Ph-IAA treatment.

We then set out to explore the utility of the AID2 system to degrade a synaptic protein in-vivo. Specifically, we injected wild type mice with a mixture of three AAV vectors in the auditory cortex: (i) hSyn-PSD95:HT:mAID to express a degradable PSD-95 variant, similar to that used for the cell culture experiments of Fig. 2A–D; (ii) hSyn-OsTIR1-P2A-NLS:tagBFP2 to allow the OsTIR1-dependent depletion of the mAID-tagged PSD-95, and (iii) hSyn-PSD95.FingR:EGFP-CCR5TC to label the exogenous as well as the endogenous PSD-95^69^. This set of vectors allowed FingR-mediated labeling of overall PSD-95 and a specific HT-mediated labeling of the exogenous, potentially degradable PSD-95 variant. After a period of three to four weeks to reach stable expression labels, mice were either injected intraperitoneally with saline (sham group, *n* = 3) or with 10 mg/kg 5-Ph-IAA (5-Ph-IAA group, *n* = 4). Six hours after treatment, animals were sacrificed, their brains were extracted and fixed. Subsequently, brain slices were prepared, stained with the HaloTag ligand JF635-HT^66^ and mounted for confocal imaging (Fig. 4A; see Methods). As shown in Fig. 4B, all fluorescent markers showed robust expression. As expected, in slices from the sham group, PSD-95-associated FingR and HaloTag fluorophores exhibited punctate expression patterns with substantial overlap, likely corresponding to excitatory synapses. Importantly, in slices from the 5-Ph-IAA treated animals, JF635-HT fluorescence was significantly reduced to approximately 60% of the signal intensity of the fluorescence range between sham treated and non JF635-HT stained control slices (*p* < 0.01; Fig. 4B, C). This drop in fluorescence concurs with the ~50% reduction in PSD-95:HT:mAID observed in culture after 6 h (Fig. 2D). In contrast, treatments with 5-Ph-IAA only modestly affected the PSD-95 FingR signal (Fig. 4D), indicating that endogenous PSD-95 levels were largely unaltered.

These experiments thus confirmed the effective depletion of a cytosolic protein and a synaptic protein in neurons in vivo using AID2 technology.

### POI degradation kinetics: dependence on expression levels

The variability of POI and OsTIR1 expression levels among neurons provided an opportunity to evaluate degradation rate dependence on mAID fusion protein and OsTIR1 expression levels. For these and subsequent experiments (see below) we created a new OsTIR1(F74G) expression vector (OsTIR1-P2A-mCherry) in which mAID:EGFP was replaced with mCherry (without the mAID degron, but still separated from OsTIR1 by a P2A sequence) which was coexpressed with PSD-95:mTurq2:mAID and exposed to 5-Ph-IAA as described above. We then measured, on a neuron by neuron basis, the initial rate of synaptic PSD-95:mTurq2:mAID degradation as a function of (i) initial PSD-95:mTurq2:mAID fluorescence, and (ii) mCherry fluorescence, the latter serving as a proxy for OsTIR1 expression levels. Initial rates of synaptic PSD-95:mTurq2:mAID degradation were estimated as linear fits to PSD-95:mTurq2:mAID fluorescence during the first 5 time points following 5-Ph-IAA addition. As shown in Supplementary Fig. 4A, PSD-95:mTurq2:mAID degradation rates (in absolute fluorescence units per h) correlated well with PSD-95:mTurq2:mAID expression levels (*r* = 0.75), as might be expected, and also with OsTIR1 expression levels (*r* = 0.65; Supplementary Fig. 4B). This sensitivity to OsTIR1 expression levels probably explains some of the variability in the degradation curves of individual neurons. It also indicates that OsTIR1 expression level is a limiting factor that dictates POI degradation rates. Finally, the linear dependence of PSD-95:mTurq2:mAID loss rate on PSD-95:mTurq2:mAID expression levels argues against a saturation of degradation capacity at the expression levels reached here. Consequently, the extent of POI loss effects might be expected to scale with the ratio of exogenous to endogenous POI copy numbers.

### Acute degradation of synaptic scaffold proteins leads to receptor loss at the same synapses

The fusion proteins of PSD-95, GKAP and Gephyrin described above were expressed over a background of endogenous variants of the same proteins. It thus remains possible that the presence of this endogenous population will mask potential consequences of (exogenous) POI loss, questioning the utility of the slow additive, rapid subtractive approach offered here for investigating synaptic POI function. We thus first considered the well-documented roles of PSD-95^51^ and Gephyrin^61^ in retaining neurotransmitter receptors at postsynaptic membranes, examining whether the rapid degradation of exogenous PSD-95 and Gephyrin is followed by substantial receptor loss from the same synapses.

**Fig. 5:**
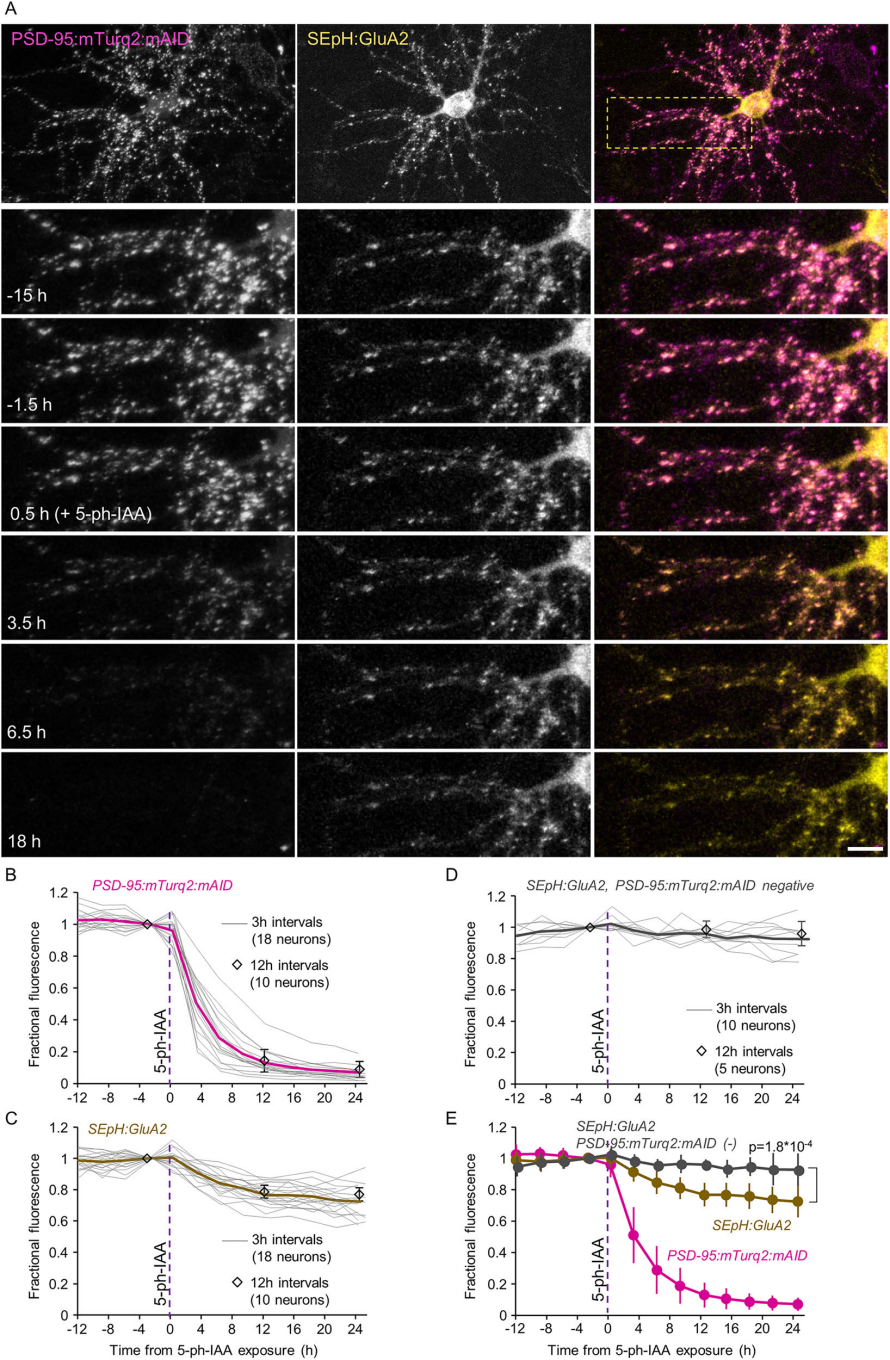
Acute degradation of PSD-95:mTurq2:mAID is followed by loss of AMPARs at the same synapses. **A** Top panels: A rat cortical neuron in culture co-expressing PSD-95:mTurq2:mAID, SEpH:GluA2 and OsTIR1-P2A-mCherry. Bottom panels: Region in yellow rectangle at greater detail, before, and after addition of 5-Ph-IAA. Note that the near complete loss of PSD-95:mTurq2:mAID fluorescence is associated with a noticeable reduction in SEpH:GluA2 fluorescence. Scale bars: 10 µm. **B** PSD-95:mTurq2:mAID fluorescence measured at 16–41 synapses of each neuron (455 in total) tracked throughout the experiments. Each thin gray line is the average fluorescence measured for the synapses of one neuron (18 neurons from 3 experiments). Fluorescence was normalized to fluorescence measured at time point just before 5-Ph-IAA addition. Thick magenta line is the population average. A subset of neurons was imaged only once every 12 (instead of 3) h to minimize potential confounds related to photobleaching (open diamonds; 10 neurons from the same 3 experiments). **C** Changes in SEpH:GluA2 fluorescence at the same synapses and neurons of (**B**). Thick brown line is the population average. **D** SEpH:GluA2 fluorescence measured at 14–30 synapses of neurons positive for SEpH:GluA2 and OsTIR1-P2A-mCherry but negative for PSD-95:mTurq2:mAID (222 in total). Each thin gray line is the average fluorescence measured for the synapses of one neuron (10 neurons from 3 experiments). Thick gray line is the population average. Open diamonds represent measurements made in a subset of cells imaged only once every 12 h (5 neurons from the same experiments). **E** Pooled data. All error bars are standard deviations, not SEM. Test for difference between PSD-95:mTurq2:mAID positive and negative cells – unpaired *t*-test, without assuming equal variances; applied to data obtained at last time point.

We first examined whether and to what degree the acute degradation of exogenous PSD-95 is followed by a loss of AMPA type ionotropic glutamate receptors (AMPARs) from the same synapses. To visualize AMPARs, we expressed a fusion protein of GluA2 and Super Ecliptic pHluorin (SEpH; e.g. refs. ^70,71^) previously used in our hands^72^ (SEpH:GluA2). Such fusion proteins effectively report outward facing AMPARs located in the neuronal membrane, as the fluorescence of SEpH within typically acidic intracellular organelles is quenched. For these experiments, we used OsTIR1-P2A-mCherry instead of OsTIR1-P2A-mAID:EGFP:NES to avoid spectral overlap with SEpH. We then triple expressed PSD-95:mTurq2:mAID, SepH:GluA2 and OsTIR1-P2A-mCherry in cortical neurons in primary culture, and followed individual postsynaptic sites as described above, before and after addition of 5-Ph-IAAPh-IAA. As shown in Fig. 5A, PSD-95:mTurq2:mAID and SepH:GluA2 puncta exhibited excellent colocalization, with comparisons of PSD-95:mTurq2:mAID and SepH:GluA2 on a synapse to synapse basis revealing a high correlation between the two (*r* = 0.64; *p* = 7.6*10^−55^; 455 synapses from 18 neurons from 3 experiments). The addition of 5-Ph-IAAPh-IAA and the consequential loss of PSD-95:mTurq2:mAID was associated with a ~25% loss of SepH:GluA2 fluorescence on average (Fig. 5C, E), with loss kinetics closely following those of PSD-95:mTurq2:mAID. As SepH:GluA2 tends to photobleach, we also measured SepH:GluA2 (and PSD-95:mTurq2:mAID) in some neurons only once every 12 h; the degree of SepH:GluA2 fluorescence loss, however, was nearly identical (Fig. 5B–D). Moreover, comparison with neurons in the same experiments that expressed SepH:GluA2 but not PSD-95:mTurq2:mAID (Fig. 5D) confirmed that the loss of SepH:GluA2 fluorescence associated with PSD-95:mTurq2:mAID degradation was statistically significant (*p* = 1.8*10^−4^; 18 and 10 neurons, respectively). Interestingly, exposure to 5-Ph-IAA was also associated with a loss of SepH:GluA2 puncta (Supplementary Fig. 5A–C; 3% at 4 h; *p* = 0.04; 14% at 24 h; *p* = 0.0002; 15 and 11 neurons, respectively) suggesting that quantification of receptor loss based on measurements made at persistent SepH:GluA2 puncta might have underestimated the full extent of PSD-95:mTurq2:mAID degradation-associated receptor loss.

In these experiments, PSD-95:mTurq2:mAID was expressed in the presence of endogenous PSD-95, possibly explaining substantial AMPAR confinement at most of synaptic sites (Fig. 5A, C, E) following PSD-95:mTurq2:mAID degradation. To quantify the impact of PSD-95:mTurq2:mAID degradation on total PSD-95 pools, we used quantitative immunocytochemistry. We first compared synaptic PSD-95 levels in PSD-95:mTurq2:mAID expressing and naïve neurons finding that PSD-95 levels at PSD-95:mTurq2:mAID positive synapses were ~3-fold higher than those of naïve neurons (24 and 31 fields of view from 2 separate experiments; Supplementary Fig. 6). We then repeated the experiments of Fig. 5, following the 24 h period of 5-Ph-IAA exposure with fixation and immunocytochemistry, using SepH:GluA2 to locate the same synapses imaged before fixation (see Supplementary Fig. 7A, B for further details). Here we found that total synaptic PSD-95 levels following PSD-95:mTurq2:mAID degradation were not significantly different from those of naïve neurons (Supplementary Fig. 7C; 3 separate experiments; 23 and 52 fields of view, PSD-95:mTurq2:mAID expressing and naïve, respectively). Thus, even in the presence of normal complements of endogenous PSD-95, rapid degradation of exogenous PSD-95 led to readily resolvable and significant loss of AMPARs from the same synapses.

The ability to resolve concomitant changes in PSD-95:mTurq2:mAID levels (~75% of total synaptic PSD-95) and SepH:GluA2 at individual synapses, allowed us to test two alternate possibilities. 1) Degradation of PSD-95:mTurq2:mAID is followed by uniform, fractional loss of AMPARs from all synapses; 2) Receptor loss differs greatly from one synapse to another, in accordance with prior suggestions based on knockout and knockdown approaches, that AMPAR loss occurs at individual synapses in all-or-none fashion^73^. To address this question, we compared, on a synapse by synapse basis, the loss of SepH:GluA2 fluorescence to the loss of PSD-95:mTurq2:mAID fluorescence after a 15 h 5-Ph-IAAPh-IAA exposure period, using non-normalized fluorescence values, as these are expected to scale linearly with fusion protein quantity. At the population level, SepH:GluA2 fluorescence loss was positively correlated with PSD-95:mTurq2:mAID loss (*r* = 0.43, 455 synapses from 18 neurons from 3 experiments; Supplementary Fig. 8A). A similar result was obtained when the correlation was calculated separately for each neuron (0.39 ± 0.22; mean ± standard deviation, respectively). Yet at the individual synapse level, the ratio of SepH:GluA2 loss to PSD-95:mTurq2:mAID loss was quite variable, with a considerable number of synapses showing no relative loss and even some gain of SepH:GluA2 fluorescence (Supplementary Fig. 8A, D). Comparisons with PSD-95:mTurq2:mAID fluorescence signals of similar magnitude indicate that this variability is not merely measurement noise (see Supplementary Fig. 8A–C for further details). Although we observed no overt bimodality in SepH:GluA2 loss to PSD-95:mTurq2:mAID loss ratios (Supplementary Fig. 8D), these findings argue against uniform receptor loss, even at relatively short time scales.

**Fig. 6:**
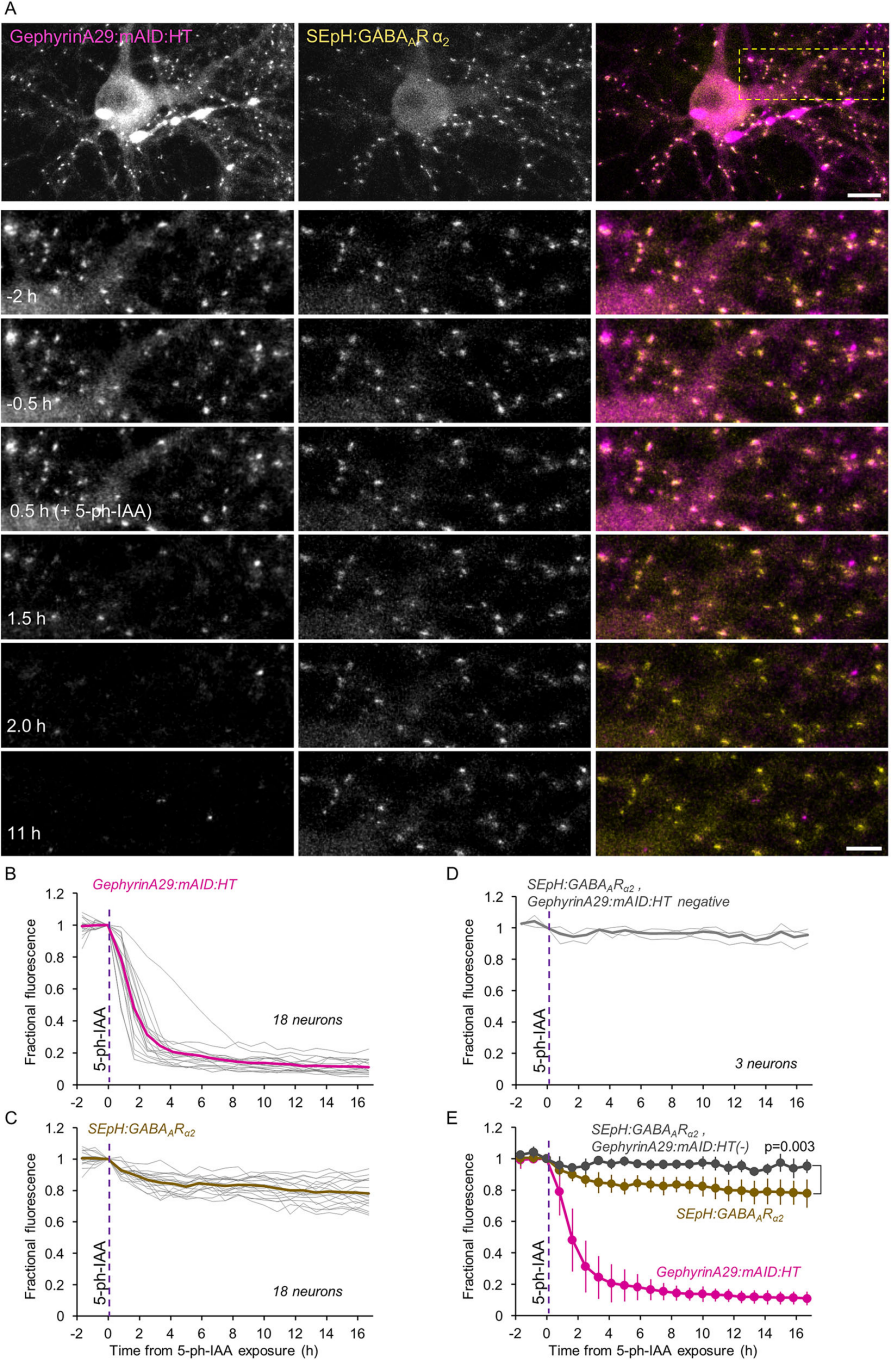
Acute degradation of GephyrinA29:mAID:HT is followed by loss of GABARs at the same synapses. **A** Top panels: A rat cortical neuron in culture co-expressing GephyrinA29:mAID:HT (labeled with JF635-HT), SEpH:GABA_A_Rα_2_ and OsTIR1-P2A-mCherry. Bottom panels: Region in yellow rectangle at greater detail, before, and after addition of 5-Ph-IAA. Note that the near complete loss of JF635-HT fluorescence (presumably reflecting GephyrinA29:mAID:HT degradation) is associated with a detectable reduction in SEpH:GABA_A_Rα_2_ fluorescence. Scale bars: 10 µm (top panels) 5 µm (bottom panels). **B** JF635-HT fluorescence measured at 50 synapses of each neuron tracked throughout the experiments. Each thin gray line is the average JF635-HT fluorescence measured for the synapses of one neuron (900 synapses from 18 neurons from 3 experiments). Thick magenta line is the population average**. C** changes in SEpH:GABA_A_Rα_2_ fluorescence at the same synapses and neurons of (**B**). Thick brown line is the population average. **D** SEpH:GABA_A_Rα_2_ fluorescence measured at 150 synapses of neurons positive for SEpH:GABA_A_Rα_2_ and OsTIR1-P2A-mCherry but negative for GephyrinA29:mAID:HT. Each thin gray line is the average fluorescence measured for the synapses of one neuron (3 neurons from 2 experiments). Thick gray line is the population average. **E** Pooled data. Error bars are standard deviations. Test for difference between GephyrinA29:mAID:HT positive and negative cells – unpaired *t*-test, without assuming equal variances; applied to data of last time point.

Similarly, Gephyrin plays key, possibly singular roles in confining GABA receptors to postsynaptic sites of inhibitory synapses^42,59–62^. We thus examined whether and to what degree the acute loss of exogenous Gephyrin is associated with GABA receptor loss at the same synapses. To that end we triple expressed GephyrinA29:mAID:HT, OsTIR1-P2A-mCherry, and a fusion protein of GABA_A_ receptor subunit α_2_ and Super Ecliptic pHluorin (SEpH:GABA_A_Rα_2_) previously shown to localize well to GABAergic synapses in cultured neurons and in vivo^74,75^. As shown in Fig. 6A, GephyrinA29:mAID:HT and SEpH:GABA_A_Rα_2_ colocalized extremely well at individual synapses. Quantitatively, comparing GephyrinA29:mAID:HT and SEpH:GABA_A_Rα_2_ on a synapse to synapse basis revealed a high correlation between the two (*r* = 0.64; *p* = 1.75*10^−105^; 900 synapses from 18 neurons from 3 experiments). We then followed individual synapses as described above, before and after addition of 5-Ph-IAA. The consequential loss of GephyrinA29:mAID:HT was associated with a ~20% reduction of SEpH:GABA_A_Rα_2_ fluorescence (Fig. 6A–D). Here too, comparison with neurons that did not express GephyrinA29:mAID:HT, revealed that receptor loss was statistically significant (*p* = 0.003). Moreover, in common with the observations made above for glutamatergic synapses, exposure to 5-Ph-IAA was associated with reductions in SEpH:GABA_A_Rα_2_ puncta counts (Supplementary Fig. 5D–F; 14% at 1.5 h; *p* = 0.002; 40% at 15 h; *p* = 0.04; 18 and 3 neurons, respectively) suggesting that quantification of receptor loss at persistent SEpH:GABA_A_Rα_2_ puncta might have underestimated the full extent of GephyrinA29:mAID:HT degradation-associated receptor loss.

As for PSD-95:mTurq2:mAID, GephyrinA29:mAID:HT, was expressed in the presence of endogenous Gephyrin, possibly explaining substantial GABA_A_R confinement at most of synaptic sites (Fig. 6A, C, E) following GephyrinA29:mAID:HT. Here too we used quantitative immunocytochemistry to quantify the impact of GephyrinA29:mAID:HT degradation on total Gephyrin pools. Here we found that total Gephyrin levels at GephyrinA29:mAID:HT positive synapses were ~1.8-fold higher than those of naïve neurons (16 fields of view in each condition, 2 separate experiments; Supplementary Fig. 9). We then repeated the experiments of Fig. 6, following the 12 h period of 5-Ph-IAA exposure with fixation and immunocytochemistry, using SEpH:GABA_A_Rα_2_ to locate the same synapses (see Supplementary Fig. 10A, B for further details), finding that here too, total synaptic Gephyrin levels following GephyrinA29:mAID:HT degradation were not significantly different from those of naïve neurons (Supplementary Fig. 10C; 3 separate experiments; 19 and 20 fields of view, GephyrinA29:mAID:HT expressing and naïve, respectively). Thus, even in the presence of normal complements of endogenous Gephyrin, rapid degradation of exogenous Gephyrin led to resolvable and significant loss of GABARs from the same synapses.

Interestingly, here too, when GephyrinA29:mAID:HT and SEpH:GABA_A_Rα_2_ loss were compared on a synapse by synapse basis, we note a positive correlation between the loss of SEpH:GABA_A_Rα_2_ and the loss of GephyrinA29:mAID:HT (Supplementary Fig. 8E). The correlation was lower, however, in comparison to that observed for excitatory synapses (Supplementary Fig. 8A), possibly due to lower overexpression levels and the use of a HT label (as compared to a fused fluorescent protein) for quantification.

Similar experiments were carried out using the N-terminally tagged Gephyrin variant mAID:mTurq2:Gephyrin. mAID:mTurq2:Gephyrin and SEpH:GABA_A_Rα_2_ also colocalized extremely well, with a very high correlation of fluorescence at individual synapses (*r* = 0.86; *p* = 4.3*10^−268^; 900 synapses from 18 neurons from 3 experiments). The loss of mAID:mTurq2:Gephyrin was associated with a ~40% reduction of SEpH:GABA_A_Rα_2_ fluorescence (Supplementary Fig. 11A–D) which closely followed the time course of mAID:mTurq2:Gephyrin degradation. Given the similar residual fractions of GephyrinA29:mAID:HT and mAID:mTurq2:Gephyrin, the greater influence of mAID:mTurq2:Gephyrin degradation on residual SEpH:GABA_A_Rα_2_ fluorescence would seem to be indicative of a greater exogenous to endogenous variant ratio for this fusion protein. Unfortunately, this could not be verified through quantitative immunocytochemistry due to the aforementioned limitations of anti-gephyrin antibodies (Supplementary Fig. 3).

These experiments thus demonstrate that even in the presence of endogenous variants of synaptic POIs, abrupt AID2-mediated degradation of overexpressed POI variants can lead to clearly detectable effects that are fully congruent with their known functions, receptor retention in this case.

### GKAP, but not PSD-95 degradation is associated with reductions in PSD sizes

As explained above, the protracted time scales of knockout, knockdown and overexpression approaches severely challenge their capacity to resolve direct influences of particular POIs on PSD organization. Conceivably, acutely degrading a particularly influential POI (a driver) would lead to PSDs shrinkage or elimination, whereas degrading less influential POIs will not. As a proof of principle, we set out to examine, in living neurons, whether rapidly reducing the synaptic contents of two prototypical PSD proteins would lead to PSD shrinkage at the same synapses. Specifically, and informed by biochemical reconstitution experiments^10,13^, we examined the consequences of rapidly degrading mAID tagged PSD-95 and GKAP.

**Fig. 7:**
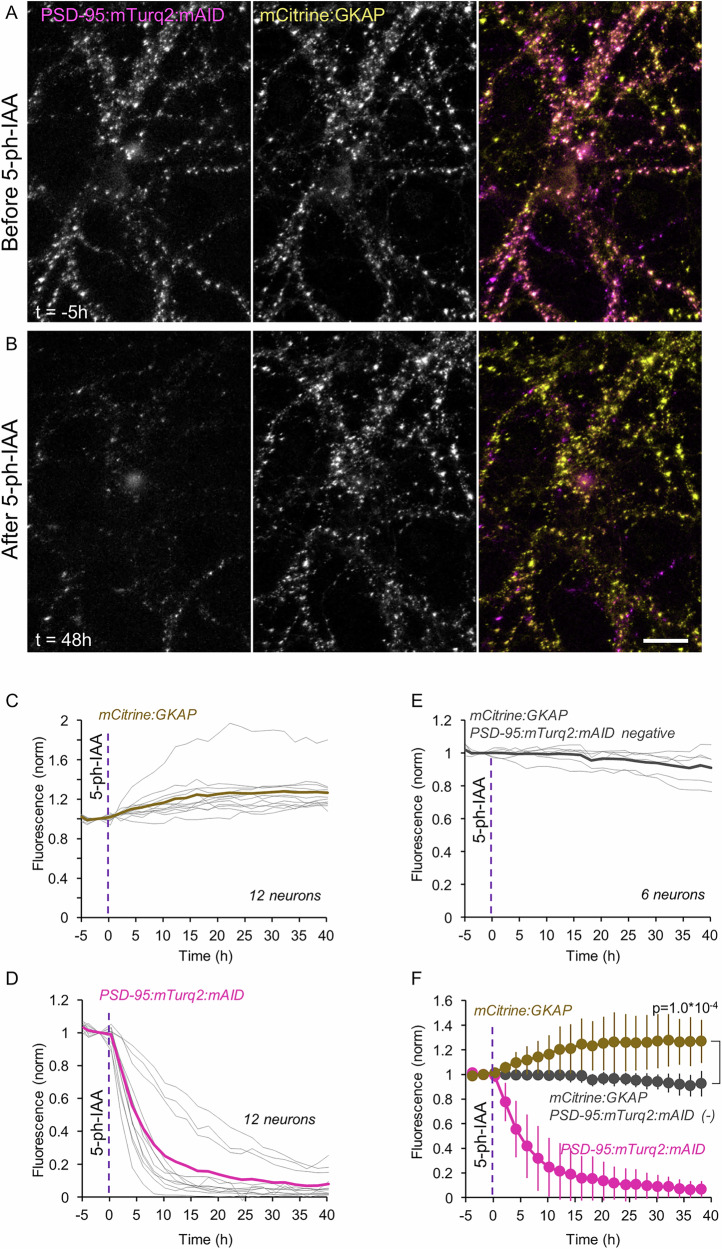
Acute degradation of PSD-95:mTurq2:mAID is followed by GKAP influx into the same synapses. **A** A rat cortical neuron in culture co-expressing PSD-95:mTurq2:mAID (left) and mCit:GKAP (middle) as well as OsTIR1-P2A-mCherry. **B** 5-Ph-IAA induced nearly complete loss of PSD-95:mTurq2:mAID, and increased synaptic levels of mCit:GKAP at the same synapses. Scale bar: 20 µm. **C** Changes in mCit:GKAP fluorescence measured at 13–40 synapses of each neuron tracked throughout the experiments (303 in total). Each thin gray line is the average normalized fluorescence measured for the synapses of one neuron (12 neurons from 2 experiments). Thick brown line is the population average. **D** PSD-95:mTurq2:mAID fluorescence measured at the same synapses and neurons of C. Thick magenta line is the population average. **E** mCit:GKAP fluorescence measured at 18–33 synapses of neurons positive for mCit:GKAP and OsTIR1-P2A-mCherry but negative for PSD-95:mTurq2:mAID (147 in total). Each thin gray line is the average fluorescence measured for the synapses of one neuron (6 neurons from 2 experiments). Thick gray line is the population average. **F** Pooled data. Error bars are standard deviations. Test for difference between PSD-95:mTurq2:mAID positive and negative cells – unpaired *t*-test, without assuming equal variances; applied to data of last time point. See also Supplementary Fig. 12.

Starting with PSD-95, we triple expressed PSD-95:mTurq2:mAID (the target), OsTIR1-P2A-mCherry and a fusion protein of GKAP and mCitrine (mCit:GKAP; the reporter) in cultured rat cortical neurons. Under baseline conditions, the correlation of mCit:GKAP and PSD-95:mTurq2:mAID fluorescence on a synapse-to-synapse basis was very high (*r* = 0.82; *p* = 7.6*10^−106^; 422 synapses from 17 cells in 3 experiments). As shown in Fig. 7, exposure to 5-Ph-IAA and the consequential loss of PSD-95:mTurq2:mAID were not associated with concomitant loss of mCit:GKAP from the same synapses (see also^57^). Instead, and somewhat unexpectedly, PSD-95:mTurq2:mAID loss was accompanied by a ~26% increase in mCit:GKAP content at the same synapses (303 synapses from 12 neurons from 2 experiments). Comparisons with neurons in the same preparations expressing mCit:GKAP but not PSD-95:mTurq2:mAID revealed that the increase in mCit:GKAP fluorescence was highly significant (*p* = 1.0*10^−4^; 12 and 6 neurons, respectively). Similar observations were made in a separate set of experiments, using synapses segmented automatically at each time point, rather than individually tracked ones (Supplementary Fig. 12; 13 neurons and 9 control neurons, from 3 experiments, >8000 synapses measured at each time point). These data indicate that in these experiments, PSD-95 was not the sole factor setting the size of these PSDs, and that its loss was followed by an influx of other scaffold proteins, in particular GKAP into PSDs. Indeed, when we co-expressed a fusion protein of PSD-95 and mCitrine (PSD-95:mCit) as a reporter instead of mCit:GKAP, we observed a nearly identical phenomenon, namely the influx and coalescence of cytosolic PSD-95:mCit at sites vacated of PSD-95:mTurq2:mAID (Supplementary Fig. 13). This influx was associated with a notable decrease in cytosolic PSD-95:mCit fluorescence (compare Supplementary Fig. 13A, B). Interestingly, the correlation at individual synapses of PSD-95:mTurq2:mAID and PSD-95:mCit fluorescence – identical proteins that differ only in the GFP variant they are fused to – was nearly identical (*r* = 0.82; *p* = 4.6*10^−114^; 450 synapses from 22 neurons in 3 experiments) indicating that this value is near the ceiling of such measurements in our experiments.

**Fig. 8:**
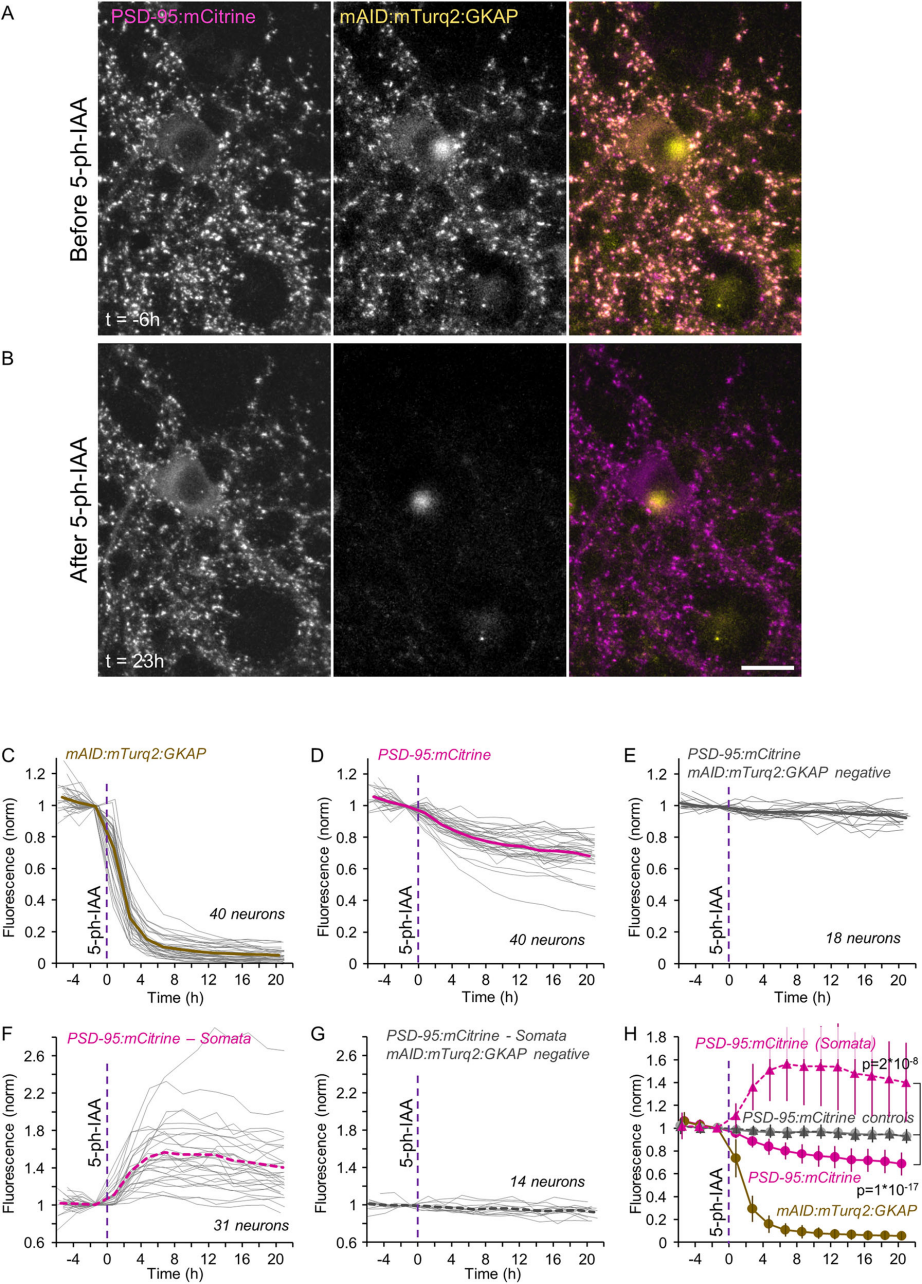
Acute degradation of mAID:mTurq2:GKAP is followed by concomitant loss of synaptic PSD-95 and elevated levels of cytosolic PSD-95. **A** A rat cortical neuron in culture co-expressing PSD-95:mCit (left) mAID:mTurq2:GKAP (middle) as well as OsTIR1-P2A-mCherry. **B** 5-Ph-IAA induced nearly complete loss of mAID:mTurq2:GKAP fluorescence, loss of PSD-95:mCit fluorescence from the same synapses and elevated levels of cytosolic PSD-95:mCit. Scale bar: 20 µm. **C** Changes in mAID:mTurq2:GKAP fluorescence measured at 24–78 synapses of each neuron tracked throughout the experiments (1723 synapses in total). Each thin gray line is the average normalized fluorescence measured for the synapses of one neuron (40 neurons from 5 experiments). Thick brown line is the population average. **D** PSD-95:mCit fluorescence measured at the same synapses and neurons of (**C**). Thick magenta line is the population average. **E** PSD-95:mCit fluorescence measured at 21–64 synapses of neurons positive for PSD-95:mCit and OsTIR1-P2A-mCherry but negative for mAID:mTurq2:GKAP (752 synapses in total). Each thin gray line is the average fluorescence measured for the synapses of one neuron (18 neurons from 5 experiments). Thick gray line is the population average. **F** Changes in cytosolic levels of PSD-95:mCit measured at the cell soma (31 neurons from 5 experiments). Dashed thick magenta line is the population average. **G** Changes in cytosolic PSD-95:mCit measured in the cell bodies of neurons positive for PSD-95:mCit and OsTIR1-P2A-mCherry but negative for mAID:mTurq2:GKAP (14 neurons from 5 experiments). Dashed thick gray line is the population average. **H** Pooled data. Dashed lines with filled triangles represent cytosolic PSD-95:mCit levels. Error bars are standard deviations. Tests for difference between PSD-95:mTurq2:mAID positive and negative cells – unpaired *t*-tests, without assuming equal variances; applied to data of last time points.

Although PSD-95 is commonly viewed as a central organizer of PSD size and properties, other studies^33,55^, including the biochemical reconstitution studies mentioned above^10,13^, pointed to the unique importance of GKAP in this regard. We thus performed the reverse experiment, that is, rapidly degraded mAID-tagged GKAP and followed the consequences to PSD-95 contents at the same synapses. To that end we triple expressed mAID:mTurq2:GKAP (the target), PSD-95:mCit (the reporter) and OsTIR1-P2A-mCherry. Here too, the correlation of mAID:mTurq2:GKAP and PSD-95:mCit fluorescence at individual synapses was maximal (*r* = 0.85; *p* = 5*10^−132^ 478 synapses from 21 cells in 3 experiments). Unlike what was observed for PSD-95, acute degradation of mAID:mTurq2:GKAP was associated with a marked reduction (~30%) of PSD-95:mCit contents at the same synapses, which closely followed the time course of mAID:mTurq2:GKAP loss (Fig. 8A–E, H; 1723 synapses from 40 neurons from 5 experiments, *p* = 9.5*10^−18^ when compared to 18 neurons that expressed PSD-95:mCit but not mAID:mTurq2:GKAP). Strikingly, the loss of synaptically associated PSD-95:mCit was associated with a parallel increase in cytosolic PSD-95:mCit levels (by ~35% when measured at the cell body; Fig. 8A, B, F–H; 31 neurons from 5 experiments, *p* = 1*10^−8^ when compared to 14 neurons that expressed PSD-95:mCit but not mAID:mTurq2:GKAP). Thus, and unlike the effects of acute PSD-95:mTurq2:mAID degradation, acute mAID:mTurq2:GKAP degradation drove an efflux of PSD-95 out of postsynaptic densities and apparent PSD shrinkage.

Comparisons on a synapse to synapse basis revealed a positive correlation between the loss of mAID:mTurq2:GKAP and PSD-95:mCit fluorescence after 15 h of exposure to 5-Ph-IAA (*r* = 0.41, 1723 synapses from 39 neurons from 5 experiments; correlations determined for each neuron separately: 0.43 ± 0.17; mean ± standard deviation, respectively). Here too, PSD-95:mCit fluorescence loss was quite variable, with some synapses exhibiting no loss and even some gain (Supplementary Fig. 8F), possibly reflecting some PSD remodeling that occurred during this time frame^52^.

These experiments demonstrate the utility of a slow additive, rapid AID2-based subtractive approach for determining the acute influences of specific postsynaptic scaffold POIs on PSD organization at individual synapses, even in the presence of endogenous variants of the same proteins.

## Discussion

Here we set out to examine the utility of an approach for studying real-time consequences of acute synaptic POI degradation at individual synapses based on AID2 technology, the coexpression of synaptic reporters and live imaging. We first established that upon expression in cortical neurons in culture, exogenous fusion proteins of PSD-95, GKAP, and gephyrin, with N-terminally, C-terminally, or intramolecularly mAID as well as a fluorescent protein (mTurq2) or a HaloTag protein, localize correctly to synapses and are rapidly degraded upon exposure to sub-micromolar concentrations of the small, membrane permeable inducer 5-Ph-IAA. Degradation was rapid, uniform and nearly complete, yet reversible upon inducer removal. We further established that the AID2 technology also allows for the rapid and reversable degradation of cytosolic and synaptic proteins in vivo, with kinetics comparable to those observed in cell culture. We found that even in the presence of endogenous variants of the same proteins, rapid degradation of exogenous PSD-95 and Gephyrin were associated with loss of AMPA and GABA_A_ receptors from the same synapses, respectively, in line with the known roles of these scaffold proteins. We then used this approach to compare the relative dominance of the two scaffold proteins PSD-95 and GKAP in determining PSD size, finding that rapid degradation of exogenous PSD-95 was associated with GKAP influx into the same PSDs and was not associated with PSD shrinkage, whereas rapid degradation of exogenous GKAP was followed by concomitant PSD shrinkage and PSD-95 efflux from the same PSDs. Altogether, these findings show that rapid degradation of exogenous variants of synaptic proteins using AID2 technology is a potentially powerful approach for studying the roles of such proteins in living neurons in situ, even in the presence of endogenous variants of the same proteins.

The main advantage of the AID2-based approach described here is found in the rapid kinetics of synaptic POI degradation, and the ability to follow the manner by which POI loss affects interacting proteins at the same synapses and at the same temporal resolution. The dramatic reduction of POI degradation times from days (knockout and knockdown approaches) to a few hours creates time windows within which direct consequences of POI loss can be studied with minimal contamination from slow adjustment/compensation processes that often complicate interpretations (e.g. refs. ^26,73^). The coexpression of fluorescently labeled interacting proteins provides real-time readouts and the full time course of consequential effects. Finally, the compressed time scales offer practical advantages in the sense that they greatly reduce the technical challenges associated with following synaptic POI degradation and consequential phenomena in situ.

The AID2 technology^25^ used here offers particular advantages. One of these lies in its simplicity: While other systems have been developed for rapidly degrading POIs, including ProTACs, molecular glue degraders^76^, GFE3^8^, PFE3^9^ and peptide-directed lysosomal degradation^77^, these typically involve specific molecules tuned to target specific POIs. Unfortunately, the identification of such molecules is non-trivial, usually involving combinations of rational design and screening. Moreover, their molecular specificity can be imperfect. Thus, for example, PFE3^9^ is based on FingRs (Fibronectin intrabodies generated with mRNA display) that bind to PSD-95, but probably also to other PSD molecules (SAP-97, SAP-102) with relatively high affinity^69^. In contrast, AID2 involves a generic degron that can be used to selectively and reliably target diverse proteins, with our data indicating that degron location along the polypeptide chain is quite flexible. We note, however, that this is also a potential disadvantage, as the same generic degron, in common with other modifications to the amino-acid sequence of a protein, could have unexpected consequences on POI function.

A second advantage is the inherent reversibility of AID2-based degradation. In this respect, new genetic approaches (e.g. ref. ^78^) also offer reversable gene manipulation. Yet, the simplicity of the AID2 system induction (and termination) mechanism - a small molecule that is easily introduced and removed or cleared, both in culture and in vivo, would seem advantageous compared to the complex molecular machineries of reversable genetic approaches. A final, although not exclusive, advantage is the systems three component composition, with each component potentially controlling a separate experimental dimension: the mAID fusion protein (what), OsTIR1 (where) and 5-Ph-IAA (when).

While AID2-based POI degradation kinetics are rapid in comparison to typical turnover rates of proteins involved in synaptic organization, (exogenous) POI loss is not instantaneous. Time constants of synaptic POI degradation in these experiments were on the order of a few hours, which might be too long for studying short-lived proteins that act on much shorter time scales. Our findings suggest, however, that kinetics can be tuned by controlling the expression levels of OsTIR1 (Supplementary Fig. 4B) and by the choice of fluorescent reporter fused to POIs, given our observation that degradation kinetics of HaloTag fusion proteins (conjugated to fluorescent ligands) were slower than those of comparable GFP-based fusion proteins.

In the experiments described here, mAID-POI fusion proteins were expressed on a background of endogenous variants of the same proteins, and thus, while exogenous POI variants were nearly completely degraded upon exposure to 5-Ph-IAA, endogenous variants remained present at more or less normal levels (Supplementary Figs. 7, 10). Estimates based on immunolabeling indicated that PSD-95 and gephyrin overexpression levels were ~3 and 1.8-fold as compared to untreated neurons indicating that exposure to 5-Ph-IAA resulted in the loss of ~75% and ~45% of total levels of these POIs. Yet, their high initial expression levels in absolute terms, undoubtedly had some impact on PSD composition as shown by many prior studies^34–41^. It is important to note, however, that this impact is a manifestation of slow reorganization processes that take place over >2 weeks, mediated by proteins that interact with the overexpressed POI, dictated in turn, by the specific properties of the POI in question. Accordingly, the consequences of its acute degradation would be expected to expose these very same interactions. Indeed, the rapid degradation of exogenous PSD-95 and gephyrin was associated with loss of AMPA and GABA_A_ receptors from the same synapses, in accordance with their known roles in this regard.

In principle, the optimal manner to use this approach and avoid the aforementioned overexpression issues would be to create knock-in animals in which the genomic loci encoding for both the POI and the reporter protein are modified to include the degron and the fluorescent proteins, crossing these with animals expressing OsTIR1 from specific promotors, or introducing OsTIR1 using viral vectors, for example. Indeed, this possibility was very recently demonstrated^45,68^. Unfortunately, even with the advanced genome editing tools available today, creating suitable mammalian models is slow, challenging the utility of such genetic models for studying diverse sets of synaptic POIs, in particular when wishing to additionally express real-time reporters consisting of known interacting proteins. Furthermore, the creation of such animals does not negate potential confounds related to POI modifications (e.g. adding degrons and fluorescent reporters). Thus, in common with all experimental approaches involving interventions (e.g. knockin, knockout, knockdown, overexpression, fusion proteins, fluorescent and affinity tags) the approach offered here has both advantages and caveats that need to be considered. In this respect, testing in cell culture the utility of this approach for studying particular POIs and optimizing tagging (e.g. tag insertion sites) is a logical, necessary and informative prerequisite to the creation of genetically modified animals.

The consequential loss of AMPA and GABA receptors, following the rapid degradation of mAID-tagged variants of PSD-95 and Gephyrin, respectively (Figs. 5, 6, Supplementary Figs. 5, 7, 10, 11) was expected, and is in line with prior studies using photoinactivation of PSD-95^7^ and elimination of gephyrin^8^ and PSD-95^9^ using fusion proteins of FingRs and E3 ligases. As mentioned above, receptor loss was incomplete, possible due to the presence of endogenous variants of the same proteins. This might indicate that receptor sequestration by endogenous scaffold proteins is already quite close to saturation (but see e.g. refs. ^26,30,35,37,79–82^). It cannot also be ruled out that the fluorescent tags and/or the exogenous degron affect scaffold protein function, which might have been the case for mAID:mTurq2:Gephyrin. We note, however, that knock-in mice expressing PSD-95-EGFP^83^, PSD-95-HaloTag^84,85^ as well as Gephyrin-RFP^67^, fusion proteins similar to those used here, are viable and do not exhibit overt phenotypical defects.

Alternatively, the degeneracy of PSD proteins, in particular at excitatory synapses^26,73^, might limit the impact of removing a single PSD scaffold protein species on receptor sequestration at postsynaptic membranes, in particular when this impact is studied following slow manipulations. Indeed, genetic elimination of PSD-95 reduces, but does not abolish the presence of AMPARs at synapses^26,29–31,49,86^. By contrast, the aforementioned study using a FingR based approach to rapidly degrade endogenous PSD-95^9^ reported a ~90% loss of synaptic GluA1 within 48 h. In spite of some uncertainties regarding the specificity of this manipulation (see above), it signifies the importance of acute approaches for uncovering roles of particular proteins in synaptic organization and function.

In contrast to the expected loss of AMPA and GABA receptors, the differential consequences of exogenous PSD-95 and GKAP degradation were less expected. While these findings are generally in line with biochemical reconstitution experiments pointing to the unique role of (phosphorylated) GKAP in condensate formation and fusion^10,13^, the observation that acute degradation of exogenous of PSD-95 was associated with the influx of GKAP (and degron-free PSD-95) into the same PSDs (Fig. 7, Supplementary Fig. 11) is not an obvious corollary of such biochemical experiments. One possible explanation is that scaffold size is determined collectively by a large number of synaptic protein species and that the loss of one of these does not necessarily reduce scaffold size, only its structure and composition, creating ‘openings’ for the binding of other proteins (see for example^31^). Indeed, complete knock out of PSD-95 does not impact dendritic spine volume in the mouse hippocampus^49^. Alternatively, competition among mutually exclusive forms of PSD-95-containing condensates (e.g. SynGAP-PSD95 and Stargazin-PSD-95^87^) might break down upon exogenous PSD-95 degradation, allowing incorporation of GKAP or GKAP containing condensates into these same PSDs^13^. Finally, these observations may reflect the temporal order of the manipulations, that is, the fact that AID2-mediated PSD-95 (or GKAP) degradation followed a protracted process of PSD growth driven by PSD-95 (or GKAP) overexpression. It is quite possible that this protracted growth is associated with ‘consolidation’ processes that stabilize synaptic sizes such that these no longer strongly depend on the original driving force (overexpression). By way of comparison, dendritic spine enlargement driven by glutamate uncaging was shown to first drive the formation of elaborate actin-based cytoskeletal scaffolds that set the stage for delayed, apparently passive incorporation of PSD-95 and other scaffold molecules (see for example^88,89^). If similar mechanisms also act during slow postsynaptic growth, rapid PSD-95 loss would not be expected to reduce postsynaptic size, at least not immediately. Interestingly, PSD protein condensates were reported to directly promote actin network formation (via Homer family members), which, in turn, would be expected to stabilize enlarged synapses^10,18^. This would be in line with the possibility that synaptic enlargement driven by scaffold protein overexpression also undergoes a consolidation process of sorts mediated by cytoskeletal remodeling. In this regard, GKAP seems to exhibit unique properties in comparison to other PSD molecules, not only because of its distinct importance to PSD protein condensate formation^10^ but also to the crucial role it plays in coupling membrane proximal PSD layers to distal, cytoskeleton-associated ones^13^. Regardless of mechanistic details, the rapid influx (Fig. 7, Supplementary Fig. 12) and efflux (Fig. 8) of PSD scaffold proteins following selective degradation of other scaffold proteins demonstrates that PSD reorganization can also occur on time scales of hours, reinforcing the notion that synapses are not so much structures as they are multimolecular assemblies, possibly organized as phase-separated condensates^24^, made of dynamic molecules that move in, out and between synaptic sites^1^.

In sum, the current study illustrates the utility of a slow additive, rapid subtractive approach based on AID2 technology in combination with fluorescent reporters and live imaging to study the roles played by specific scaffold proteins in synaptic organization, and potentially for deciphering the specific roles many other synaptic proteins play in synaptic function, plasticity and tenacity.

## Methods

### Animals and cell culture preparations

Primary cultures of cortical neurons were prepared from newborn Wistar rats (either sex), in compliance with all relevant ethical regulations for animal use and following protocols approved by the Technion Israel Institute of Technology’s Committee for the Supervision of Animal Experiments (approval IL-105-08-20). In brief, cortices from 0 to 1-day-old rats were dissected and dissociated using trypsin, followed by gentle trituration with a siliconized Pasteur pipette. Approximately 5 × 10^5^ of dissociated cells were then plated on polyethylenimine-coated 29 mm glass-bottom dishes (MatTek) to promote adherence. The neurons were initially grown in a medium consisting of Eagle’s minimal essential medium (Sigma-Aldrich), 25 µg/mL insulin (Sigma-Aldrich), 20 mM glucose (Sigma-Aldrich), 2 mM L-glutamine (Sigma-Aldrich), and 10% NuSerum (Becton Dickinson Labware). Cultures were maintained in a humidified incubator at 37 °C with 95% air and 5% CO_2_. From day 7, half of the culture media was replaced three times a week with feeding medium, similar to the media described above, except for the absence of NuSerum, a reduced concentration of L-glutamine (0.5 mM), and the addition of 2% B-27 supplement (Gibco).

For the in-vivo experiments (Figs. 3, 4), wildtype C57BL/6J mice were used. At the time of starting the experiments, all animals were at ages of six to eight weeks. For the experiments of Fig. 3 a total of 3 mice were used (each iteratively underwent multiple treatment conditions). For the experiment of Figs. 4, 7 mice were used that were split into two groups receiving different treatment (*n* = 4, 5-Ph-IAA, *n* = 3 sham treatment). We have complied with all relevant ethical regulations for animal use: All animal experiments were performed in accordance with the German laboratory animal law guidelines for animal research and had been approved by the Landesuntersuchungsamt Rheinland Pfalz (Approval # G 17-1-051 and G 22-1-091).

### DNA constructs

For experiments carried out in cell culture, fusion proteins were introduced using third-generation lentiviral expression vectors based on a modified FUGW (FUGWm) backbone^90^ in which an XhoI restriction site was moved to a downstream position. Full, annotated sequences of all lentiviral plasmids used here are provided as supplementary materials. Gene synthesis and cloning were done by Genscript (Piscataway NJ, USA).

Lentiviral vectors for expressing OsTIR1(F74G) in neurons in culture were created as follows: pAAV-hSyn-OsTIR1(F74G) plasmid^25^ was obtained from Addgene (Addgene #140730). Then, the Synapsin promotor and the coding region were cut out of pAAV-hSyn-OsTIR1-F74G with MluI and BclI. PacI (5’) and XhoI (3’) sites were added to the excised segment which was then inserted into FUGWm at its PacI and XhoI sites, resulting in OsTIR1-P2A-mAID:EGFP:NES. OsTIR1-P2A-mCherry was created by full length synthesis of mCherry flanked by BsmBI and BstBI and replacing the mAID:EGFP segment in OsTIR1-P2A-EGFP:mAID with this insert.

The vector encoding for SEpH:GluA2 was described previously^72^. All other plasmids used here (PSD-95:mCit, mCit:GKAP, PSD95:mTurq2:mAID, mAID:mTurq2:GKAP, SEpH:GABA_A_Rα2, mAID:mTurq2:Gephyrin, PSD-95:HT:mAID, GephyrinA29:mAID:HT) were made by large scale gene synthesis of the POI followed by insertion into the modified FUGWm backbone described above using appropriate restriction sites. All inserts as well as at least 200 flanking base pairs were sequenced and checked for correctness.

### Lentivirus production and transduction

Lentiviral particles were generated by transfecting HEK293T cells with a plasmid mixture containing essential HIV packaging genes and a heterologous viral envelope gene (MISSION® Lentiviral Packaging Mix, Sigma). Transfection was carried out using Lipofectamine 2000 (Invitrogen), with HEK293T cells raised on 10 cm plates at ~90% confluence. The supernatant was collected 48–72 h post-transfection, filtered through 0.45-μm filters, aliquoted, and stored at −80 °C. Neurons were infected with either one or more of the constructs mentioned earlier. For double or triple infections, the viral particles were mixed before they were added to the plates. The infection was done for most experiments on 9–10 post-plating, except for the experiments of the triple expression of mAID-mTurq2-GKAP1 with PSD-95:mCit and OsTIR1-P2A-mCherry in which the neurons were infected on day 3 post-plating.

### AAV production

For AAV production 6 × 10^7^ HEK 293 cells were seeded in a 16-layer Celldisc (Greiner; Cat. no. 678916) with 1 L complete growth media (DMEM, Gibco; Cat. No. 52100–047), supplemented with 10% heat-inactivated FBS (Sigma; Cat. No. F7524), 2 mM L-glutamine (Sigma; cat. no. G7513) and 1% Penicillin Streptomycin (Sigma-Aldrich Cat. No. P0781-100ML) and cultured for 48 h in CO_2_ incubator (37 °C temperature, 95% relative humidity and 5% CO_2_). For chemical transfection plasmid pADDeltaF6 (Addgene Cat. No. #112867), pAAV8 (Addgene Cat. No. # 112864) and the respective expression vector plasmid were mixed at equimolar ratio to a total of 2.069 mg DNA. 69 ml of 300 mM CaCl_2_ was added to the plasmid DNA. The entire CaCl_2_/DNA mixture was slowly added to 69 ml 2xHBS solution (Aesar; Cat. No. #J62623). After 5 min. incubation the mixture was added to 500 ml DEMEM supplemented with 5% FCS (no antibiotics). Culture media was then carefully decanted from the Celldisc and replaced with the transfection media. After 6 h incubation (37 °C, 95% relative humidity and 5% CO_2_) transfection media was carefully decanted and replaced with 1 L of fresh complete growth media. Transfected cells were incubated for 72 h (37 °C, 95% relative humidity and 5% CO_2_).

To harvest the cells growth media was carefully decanted and collected. 500 ml of kept growth media was supplemented with 7 ml 0.5 M EDTA (Invitrogen; Cat. No. #15575-020) and 400 ml out of it was put back into the Celldisc. After 5 min incubation at room temperature cells detached from the surface. Cell suspension was transferred to a 500 ml centrifugation flask (Corning; Cat. No. 431123). The remaining 100 ml Growth media/EDTA mix was used to wash the Celldisc and added to the centrifugation flask.

After centrifugation at 800 × *g* for 15 min at 4 °C, supernatant was carefully discarded. The cell pellet was resuspended in 10 ml PBS, transferred to a 50 ml Falcon tube and centrifuged again for 15 min at 800 × *g*, at 4 °C. PBS was then discarded and the pellet resuspended in 24 ml lysis buffer (50 mM Tris, 1 M NaCl, 10 mM MgCL2) supplemented with 0,001% Pluronic F-68: (Invitrogen #24040032), 1300U Salt Active Nuclease (SAN; Sarstedt #83.1803) and 100× HALT Protease Inhibitor Cocktail, (EDTA-free Thermo scientific #78439). Cell suspension was then subjected to three freeze/thaw cycles in liquid nitrogen and a 37 °C water bath, respectively. To assure that the suspension does not contain any remaining plasmid DNA, it was again supplemented with 1300U of SAN afterwards, and incubated at 37 °C for 1 h, while shaking at 150 rpm. Following centrifugation at 2500 × *g* for 15 min at r.t, cell debris was discarded, and the supernatant was transferred to a new 50 ml Falcon tube. 40% PEG-8000 solution (Polyethyleneglycol, Sigma #89510, in H2O, supplemented with 0.001% Pluronic) was added to a final concentration of 8%, mixed and incubated on ice at 4 °C for 16 h to 24 h. After centrifugation at 2500 × *g* for 30 min at 4 °C, supernatant was discarded, any residual PEG was carefully removed and 14.5 ml resuspension buffer (50 mM TRIS, 1 M NaCl, 0.001% Pluronic, pH8.0) was added to the pellet and the pellet was resuspended by vortexing and pipetting before it was incubated for at least 24 h at 4 °C, while shaking at 350–400 rpm. It was found crucial to resuspend the pellet completely. The suspension was then centrifuged at 2500 × *g* for 30 min at 4 °C and the supernatant was transferred to an ultra-centrifugation tube (Quickseal Tubes, Beckman Coulter #342414). AAV purification was performed by ultra-centrifugation over a discontinuous Iodixanol density gradient (OptiPrep Density Gradient Medium, Sigma #D-1556, 60% solution in H2O), with Iodixanol phases of 15%, 25%, 40% and 54%, respectively. After centrifugation, ~3.5 ml of the Iodixanol phases containing the filled AAV capsids were collected (2.5 ml of 40% and 1 ml of 54% phase). Special care was taken not to touch the 25% phase, since it contains empty capsids. For buffer exchange and concentration, AAV purification buffer (1× PBS, 1 mM MgCl2, 2.5 mM KCL, 0.001% Pluronic, pH 7.4) was added to the virus containing fraction, to a total volume of 12 ml and transferred to a 15 ml AMICON ULTRA-15 column; (MWCO 100 kDa, Millipore #UFC910024). Centrifugation was performed according to the manufacturers protocol. After concentration of the virus solution to ~1.5 ml, fresh AAV purification buffer was added to a total volume of 12 ml, and centrifugation was repeated. At a volume of 0.5 ml to 1 ml, virus solution was resuspended thoroughly, transferred to a new tube and stored at −80 °C. The genomic titer was determined by qRT-PCR.

### Stereotaxic injection

All surgical equipment was sterilized with 70% ethanol before use. Animals were deeply anesthetized with isofluorane (Abbott Animal Health, IL, USA; IsoFlo) and positioned in a stereotaxic frame (Kopf Instruments, Tujunga, CA, USA; Stereotaxic System Kopf 1900). The eyes were protected from dehydration and intensive light exposure using Vaseline and a piece of aluminum foil. The anesthesia was maintained by delivery of a 1.5–2.4% isoflurane/air mixture with a vaporizer (High Precision Instruments, MT; Univentor 400 Anaesthesia Unit) at a flow rate of around 200 ml/min to the snout. Lidocaine was applied as local anesthetic subcutaneously before exposure of the skull. The scalp was washed with a 70% ethanol solution and a cut along the midline revealed the skull. A small hole was drilled into the skull above the auditory cortex using a motorized dental drill, leaving the dura mater intact. Injections were performed perpendicular to the surface of the skull. Virus solutions were specific for each experiment and consisted of a mixture of different AAV viruses in PBS: For the in-vivo experiments of Fig. 3, a 1:1 mix of AAV8-hSyn-OsTIR1(F74G)-P2A-mAID:EGFP:NES^25^ Addgene #140730, titer: $1.4* {10}^{15}$ VG/mL) and AAV8-phSyn-H2B::mCherry (titer: $1.8* {10}^{14}$ VG/mL) was injected. For the in-vivo experiments of Fig. 4, a 1:1:1 triple-mix of AAV8-hSyn-PSD-95:HT:mAID (approx. titer: ${10}^{13}$ VG/mL), AAV8- hSyn-OsTIR1-P2A-NLS:tagBFP2 (approx. titer: ${10}^{13}$ VG/mL) and AAV8-hSyn-PSD-95.FingR:EGFP-CCR5TC (titer: ${{{\mathrm{1,0}}}* 10}^{15}$ VG/mL) was injected. The virus mixture was loaded into a thin glass pipette and 170 nl were injected at a flow rate of 20 nl/min (World Precision Instruments, Sarasota, FL, USA; Nanoliter 2000 Injector) in five locations of the right auditory cortex, resulting in a total injection volume of 850 nl. Stereotactic coordinates were: 4.4, −2.5/−2.75/−3/−3.25/−3.5, 2.5 (in mm, lateral, caudal, and ventral in reference to Bregma). Glass pipettes (World Precision Instruments, Sarasota, FL, USA; Glass Capillaries for Nanoliter 2000; Order# 4878) had been pulled with a long taper and the tip was cut to a diameter of 20–40 μm. After the injection, the pipette was left in place for three minutes, before being slowly withdrawn and moved to the next coordinate. After completion of the injection protocol, the skin wound was sealed using tissue adhesive (3 M Animal Care Products, St. Paul, MN, USA; 3 M Vetbond Tissue Adhesive), and anesthesia was terminated. Mice were monitored daily and intraperitoneal injections of carprofen (0.2 ml of 0.5 mg/ml stock) were applied on the first days after surgery.

### Cranial window implantation

Four to eight weeks after stereotactic injections, animals were anesthetized using isoflurane (Abbott Animal Health, IL, USA; IsoFlo). All surgical equipment and glass cover slip were sterilized with 70% ethanol before use. Anesthesia was initialized in a glass desiccator filled with an isoflurane/air mixture. Anesthetized animals were mounted on a stereotaxic frame (Kopf Instruments, Tujunga, CA, USA; Stereotaxic System Kopf 1900) and the head was positioned using ear, teeth, and a custom-made V-shaped head holder. Anesthesia was maintained by delivery of a 1.5 to 2.4% isoflurane/air mixture with a vaporizer (High Precision Instruments, MT; Univentor 400 Anaesthesia Unit) at a flow rate of around 200 ml/min to the snout. 0.02 ml dexamethasone (4 mg/ml) was administered intramuscularly to the quadriceps, as well as 0.02 ml carprofen (0.5 mg/ml) intraperitoneally. The eyes were protected from dehydration and intensive light exposure using Vaseline and a piece of aluminum foil. Lidocaine was applied subcutaneously before exposure of the skull. The scalp was washed with a 70% ethanol in water solution and a flap of skin covering temporal, both parietal regions and part of the occipital bone was removed. All following surgery steps were conducted unilaterally on the right side of the animal: The temporal muscle was partly removed with a surgical scalpel and forceps to expose the right temporal bone. Using a fine motorized drill, the bones of the skull were smoothened, and part of the zygomatic process was removed. The surface was cleaned using cortex buffer and covered with a thin layer of one component-instant glue (Carl Roth, Germany; Roti coll). A thin layer of dental cement (Lang Dental, IL, USA; Ortho-Jet) was applied onto the skull, sparing the area of the temporal bone above the auditory cortex. An elliptic groove of about 3 mm diameter was carefully drilled into the skull above the auditory cortex, and the bone was carefully lifted using scalpel and forceps. The exposed area was carefully cleaned and kept moist using sterile sponge (Pfizer, NY, USA; Gelfoam) and cortex buffer. The craniotomy was covered with a small circular cover glass (Electron Microscopy Sciences, PA, USA; five mm diameter, catalogue #72195-05. The cover glass was finally set in place with one component-instant glue and dental cement. In order to position the animal under the microscope with the objective facing the window plane perpendicularly, a custom-made titanium head post was mounted on the implant above the window and embedded with dental cement. After dental cement had dried, animals were placed back in a pre-warmed cage. After the surgical procedure, animals recovered for at least one week before continuing the experiments.

### Confocal imaging (cell culture experiments)

Glass-bottom dishes containing cortical neurons expressing one or more of the previously mentioned fusion proteins were imaged on a custom-built confocal laser scanning (inverted) microscope based on the Zeiss Axio Observer Z1, equipped with a 40×, 1.3 N.A. Plan-Fluar objective. The system, controlled by custom software, was designed to allow automated, multisite time-lapse microscopy. Dishes were fitted with a custom-designed cap featuring inlet and outlet ports for perfusion and air exchange. Neurons were continuously perfused with feeding media (described above) starting 24 h before imaging commenced and continuing until the conclusion of the experiment. The perfusion feeding media was supplemented with 7–10% distilled water to compensate for evaporation and delivered to the dish at a flow rate of ~3 ml/day. This was achieved using custom perfusion systems based on ultraslow-flow peristaltic pumps (Instech Laboratories, Inc.) and silicone tubing, connected to the dish via ports in the cap. A sterile gas mixture of 95% air/5% CO_2_ was delivered into the dish at low rates, regulated by a high-precision flow meter (Gilmont Instruments), either directly to the dish through a dedicated inlet in the cap or using a closed chamber above the dish. The objective of the microscope and the base of the headstage were heated to 37 °C using resistive elements with separate temperature sensors and controllers, maintaining the culture medium at 35–36 °C.

Time-lapse imaging was typically performed by averaging five frames at 10–15 focal planes, spaced 0.8 µm apart. Images were captured at a resolution of 640 × 480 pixels, and 12 bits per pixel. Images were collected sequentially from multiple sites using a motorized stage that cycled automatically through locations at predetermined intervals. Focal drift was automatically corrected using the autofocus system of the microscope.

Fluorescent proteins and labels were imaged using the following excitation sources and emission filter combinations: mTurquoise2 – 457 nm excitation (Argon laser, National Laser Company), FF01-475/20 or FF01 483/32 (Semrock), emission; EGFP and pHluorin – 488 nm excitation (Argon laser), ET 525/50 (Chroma) or FF03 525/50 (Semrock), emission; mCitrine – 514 nm excitation, (Argon laser), HQ545/50 (Chroma), emission; mCherry – 594 nm excitation (DPSS laser; Cobolt), BLP01 594R (Semrock), emission; JF552-HT– 552 nm excitation (DPSS laser; Coherent), ET 590/50 (Chroma), emission; JF635-HT and Alexa 647/Cy5 decorated (secondary) antibodies – 632 nm excitation (Helium Neon laser; JDS Uniphase/Thorlabs), RET 638 (Chroma) or LP02-633RU (Semrock), emission.

### Two-photon imaging (in vivo experiments)

The two-photon microscope (Prairie Technologies, WI, USA; Ultima IV) was comprised of a 20x-objective (Olympus, Tokyo, Japan; XLUMPlan Fl, NA = 0.95) and a pulsed laser (Coherent, CA, USA; Chameleon Ultra). All fluorophores (EGFP and mCherry) were co-excited at 920 nm wavelength, and separated by emission using a fluorescence filter cube (filter one: BP 480–550 nm; filter two: LP 590 nm; dichromatic mirror: DM 570 nm; Olympus, Tokyo, Japan; U-MSWG2). For the recordings, mice were lightly anaesthetized with isoflurane (flow from 0.9 to 1.6% isoflurane/air as described above) in order to avoid movement artifacts. Line scan imaging was performed using a field of view of 367 × 367 μm (1024 × 1024 pixels) at a sampling period of 3.024 s per frame. For each mouse, and imaging session, one to three spots from the cortical surface were imaged, where for each spot a z-stack of 70 images was recorded with a step size of 2 μm in between. For each spot, the z-level of the dura mater was identified and then, the image stack was recorded at a depth of 50–190 μm below the dura. For longitudinal tracking of the same cortical spots, the microscopic blood vessel patterns on the brain surface and the neuronal fluorescence patterns on superficial image planes were identified and matched to the reference recording from the first baseline time point. Animals were imaged on two baseline time points prior to any treatment (−1 and −0.5 h). Then, animals were randomly assigned to be injected with 10 mg/kg 5-Ph-IAA or PBS. In the following, the same set of cortical spots were imaged repeatedly at intervals of 1, 3, 6, 9, 24, 48 and 72 h post injection. Between imaging periods, animals were placed back in their home cages. All mice repeatedly underwent both treatments (sham and 5-Ph-IAA) with intervals of ~1 week, where we ensured that before starting a new recording round the mAID:EGFP fluorescence had fully recovered, matching the baseline conditions from the initial recording.

### 5-Ph-IAA preparation and treatment

For cell culture experiments, 5-Ph-IAA (MedChemExpress, # HY-134653) was dissolved in DMSO and stored as 100 mM aliquots at −80 °C. For use, the 5-Ph-IAA was diluted in feeding medium to a final working concentration of 200 nM.

For in vivo experiments, 5-Ph-IAA was dissolved in PBS. For the treatment, mice were injected with 120 μl of the solution intraperitoneally, corresponding to 10 mg/kg body weight. For the sham treatment, mice were intraperitoneally injected with 120 μl PBS.

### HaloTag ligands preparation, labeling and staining

The Halotag ligands used in this study were Janelia Fluor® 635 HaloTag® Ligand (JF635-HT) and Janelia Fluor® 552 HaloTag® Ligand (JF552-HT)^66^, both were provided as generous gifts by Luke Lavis, Janelia Research Campus.

For the cell culture experiments, the ligands were dissolved in DMSO and kept as 100 µM aliquots stocks at −20 °C. The ligands were added to the dishes 1 h before the beginning of the experiment, at a final concentration of 100 nM. To assess the recovery of the tagged proteins following the wash, a second labeling with the ligands was performed.

For labeling brain slices, a stock solution of JF635-HT with DMSO, (D2650, Sigma Aldrich), Pluronic F-127, and sterile PBS^85^ was prepared. From this stock solution, a diluted working solution of 400 nM JF635-HT in PBS was prepared just prior to addition to slices. This dye solution was added to the brain slices, followed by incubation at RT for 2 h. Finally, the dye was removed and the slices were washed two times for 15 min with PBS. Sections were mounted with Fluoromount-G.

### Preparation and imaging of histological samples

For the experiments of Fig. 4, mice were sacrificed via cervical dislocation 6 h after treatment with 5-Ph-IAA or PBS respectively. The brains were extracted, briefly cleaned in PBS and then transferred in 4% paraformaldehyde (PFA) in PBS. The brains stored in 4% PFA at 4 °C for 24 h, before re-transferring them into PBS. Slices of 70 μm (coronal section) were obtained using a Leica VT1000S vibratome.

Images of the cortical regions were acquired with a 100× oil objective (Leica; HC PL APO CS2, NA = 1.4) using Stellaris 8 FALCON confocal microscope (Leica; funded by the Deutsche Forschungsgemeinschaft, project number 497669232). Field of views of 116.25 × 116.25 μm (1664 × 1664 pixels) at layer2/3 of cortex are taken. Lasers 405, 488, and 640 were used to excite tagBFP, eGFP, and JF635 signals respectively. All channels were acquired in counting mode using line accumulations at speed of 400.

### Immunocytochemistry

Cultures of cortical neurons (naive or infected) were stained as follows: Cells were washed with Tyrode’s physiological solution (119 mM NaCl, 2.5 mM KCl, 2 mM MgCl_2_, 25 mM HEPES; 30 mM Glucose and 2 mM CaCl_2_, pH 7.4) and exposed to a fixative solution (4% formaldehyde and 4% Sucrose in PBS) for 20 min at room temperature. This was followed by adding fixative solution supplemented with Triton X-100 (0.25%) for an additional 20 min, followed by washing with PBS. Fixed cells were then incubated with 10% bovine serum albumin (BSA) in water for 1 h at 37 °C. Following this blocking step, the cells were stained overnight at 4 °C with FluoTag®-X2 anti-PSD95 Alexa 647 (NanoTag Biotechnologies # N3702-AF647-L; 1:200), or exposed to a mouse anti gephyrin primary antibody (Synaptic Systems anti gephyrin #147 111 or #147 011; 1:500 and 1:1000, respectively) overnight at 4 °C, followed by washing with PBS and labeling with a secondary antibody (Cy™5 AffiniPure conjugated polyclonal Donkey Anti-Mouse IgG; Jackson ImmunoResearch Laboratories, #715-225-150) (1:200) 1 h at 37 °C. Cells expressing GephyrinA29:mAID:HT were labeled with the HT ligand JF552-HT prior to fixation.

### Image processing and analyses

All imaging data collected in cell culture were analyzed using custom software (‘OpenView’), which offers both automated and manual tracking of individual objects as well as measurements of fluorescent intensities over time. For fluorescence intensity measurements of synapses, regions of interest (ROIs; 9 × 9 pixels) were programmatically placed on fluorescent punctate objects at the initial time point and then semi-automatically tracked over time, focusing only on synapses that persisted throughout the experiment (synapses that split, morphed, merged, formed or disappeared were excluded from the analysis). Mean pixel intensities within these ROIs were calculated from maximal intensity projections of Z-stack sections. To assess cytosolic fluorescence levels, ROIs were manually positioned at initial time points over cell bodies, and mean pixel intensities were measured for each time point using Z-stack projections. Fluorescence values were corrected for background fluorescence by subtraction of values measured at cell-free regions, in some cases supplemented by spectral unmixing when fields of view contained substantial non-specific fluorescent objects.

In most cases, data collected from individual synapses were normalized by dividing fluorescence values collected from synapses (and somata) by values collected at the time point prior to the addition of 5-Ph-IAA.

Time constants for degradation curves were derived using a program written in Visual Basic for Applications within Microsoft Excel. The application attempts to fit the data to a sum of two exponentials, each with its own time constant, with a third parameter representing the relative fraction of each component. The application systematically explores a wide range of parameter and returns the values that result in a minimal sum of squared residuals. Time constants stated in the manuscript are for the large pools (78–98%). Time constants derived for minor pools were typically an order of magnitude longer, probably reflecting secondary processes such as photobleaching.

For the experiments of Fig. 3, the recorded image stacks from each cortical spot were fed into a custom standardized MATLAB processing pipeline^91,92^ that enabled us to track the same set of neurons from multiple defined image plains (syn. fields of view, FOV) of a stack and assess their cytosolic fluorescence signal over time (see Supplementary Fig. 14A). (i) We first defined a selection of 13 FOV to analyze per stack. Specifically, we used the initial baseline recording to select the FOV on a z-level of 60 μm below the dura mater as well as the following twelve deeper FOV in steps of 10 μm. (ii) For each recording time point, we identified the image planes that best matched these initial FOVs in respect to their nuclear H2B:mCherry signal. For this, we calculated key points on the images using a SIFT algorithm (scale-invariant feature transform) and cross-checked the key points with a brute force matching strategy (Euclidean distance-based match-validation). (iii) We then automatically identified regions of interest from the H2B:mCherry images (ROIs corresponding to nuclei). For this, images were equalized with a Gaussian blur filter, binarized (Otsu threshold) and blob detection was applied, using a Laplacian-of-Gaussian algorithm. (iv) Each FOV was then aligned to the reference image from the initial time point, applying an xy-translation and tracking the ROIs with a local affine transformation. (v) Lastly, we quantified the cytosolic fluorescence signal by defining a ring-shaped area around the centroid of each ROI (between 4–8px radius) and averaging the green fluorescence from these areas, obtaining a single-cell read-out of EGFP expression. To account for recording noise from session to session, this measure was normalized to the mean nuclear fluorescence of the H2BmCherry (see Supplementary Fig. 14A). For the analyses of the signal stability after sham/5-Ph-IAA treatment, we only included neurons with a least distance of 3px and a minimal initial normalized green fluorescence ≥0.3.

For the experiments of Fig. 4, for each image (FOV) we used a custom-written MATLAB software to manually identify representative circular ROIs on the PSD-95.FingR:EGFP and the PSD-95:HT:mAID+ JF635-HT images. Specifically, we separately estimated ROIs for neuropil areas of the image (showing no nuclei and a punctate PSD-95 signal) and background areas of the image (blood vessel areas with very low signal intensity; see Supplementary Fig. 14B). For neuropil and background respectively, we computed the mean fluorescence from all ROIs of a given FOV in both channels, PSD-95.FingR:EGFP and PSD-95:HT:mAID+ JF635-HT. Lastly, we divided the mean neuropil fluorescence by the mean background fluorescence, yielding a normalized read-out of bulk synaptic PSD-95 signals (see Supplementary Fig. 14B). We separately compared the bulk fluorescence values of the PSD-95.FingR:EGFP and the PSD-95:HT:mAID+ JF635-HT channel.

Illustrations in Figs. 3A and 4A were created in-house using Inkscape with no use of images from external repositories.

### Statistics and reproducibility

Cell culture replicates are described explicitly in the main text and figure legends as experiments (separate cell culture dishes) from separate cell culture preparations, carried out independently of each other), neurons (individual neurons within cell culture dishes) and synapses. In vivo replicates are described in the main text and figure legends explicitly in terms of individual mice, neurons and fields of view.

Comparison between pairs of experimental groups of experiments carried out in cell culture were tested using two-tailed *t*-tests without assuming identical variances using Microsoft Excel. Group differences between the auxin and sham treated animals in the in-vivo experiments were tested by applying two-sided Wilcoxon rank sum tests using the Matlab statistics toolbox software.

### Reporting summary

Further information on research design is available in the Nature Portfolio Reporting Summary linked to this article.

## Supplementary information

Supplemental Information

Supplemental Movie 1

Supplemental Data 1

Supplemental Data 2

Supplemental Data 3

Description of Additional Supplementary Files

Reporting summary

## Data Availability

Source data underlying graphs and charts presented in main figures are provided as Supplementary Data 1 (cell culture experiments) and Supplementary Data 2 (in-vivo experiments). Additional data are available from the corresponding author on reasonable request. Annotated sequences of all plasmids used here are provided in Supplementary Data 3.
