# Supplementary material for: Revealing acute consequences of rapid degradation of synaptic fusion proteins at individual synapses using Auxin-Inducible Degron 2 technology
Source: Commun Biol. 2025 Nov 17;8:1589. doi: 10.1038/s42003-025-08996-8 (PMC12623870; doi:10.1038/s42003-025-08996-8)
Supplement: Supplementary file 1 — Supplemental Information [file 42003_2025_8996_MOESM1_ESM.pdf]

Supplementary Information

Revealing Acute Consequences of Rapid Degradation of Synaptic Fusion Proteins at Individual Synapses using Auxin-Inducible Degron 2 Technology

Lilach Elbaum-Mendelson, Weixiang Yuan, Johannes P.-H. Seiler, Nadia Blom, Ya-Chien Chan, Ali Hyder Baig, Nils Brose, Simon Rumpel, and Noam E. Ziv

References mentioned in figure legends are provided at the end of the document

Supplemental Figure 1

Rapid degradation of EGFP fused to a mAID degron.

A) A vector for expressing both OsTIR(F74G) and EGFP fused to a mAID degron and a nuclear export sequence (NES), separated by a P2A sequence. Same construct as described by Yesbolatova et al., 2020, now in a lentiviral expression vector. **B)** Examples of neurons expressing OsTIR1-P2A-mAID:EGFP:NES (yellow) either exposed (right hand panels) or not exposed (left panels) to 200nM 5-Ph-IAA. Magenta dots are fluorescent background objects, used here to show that the loss of EGFP fluorescence is not a result of focal drift. Bar, 20µm. **C)** EGFP fluorescence measured at the cell bodies of 17 and 13 neurons exposed or not exposed to 5-Ph-IAA, respectively (thin gray lines). Fluorescence values for each neuron normalized to fluorescence measured at the last time point before 5-Ph-IAA was added. Thick red (5-Ph-IAA treated) and blue (untreated) lines represent population averages for each condition. Data from two separate experiments.

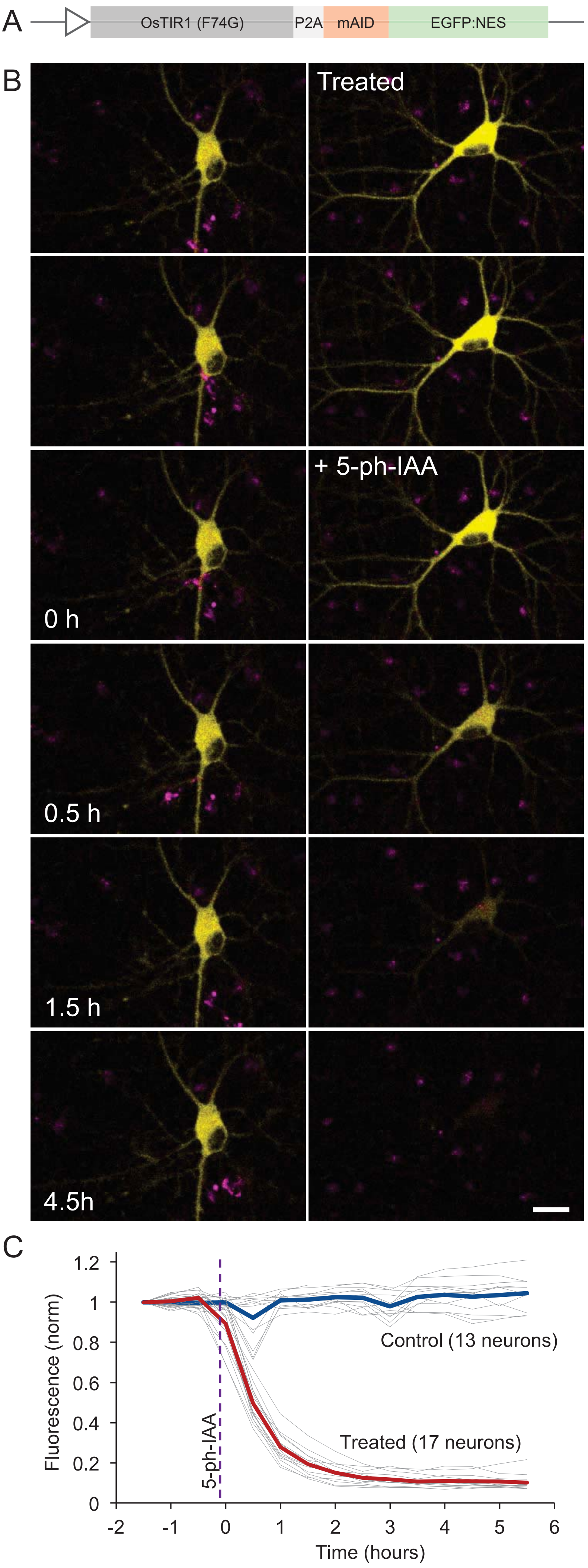
A
OsTIR1 (F74G)
P2A
mAID
EGFP:NES
B
Treated
+ 5-ph-IAA
0 h
0.5 h
1.5 h
4.5h
C
Fluorescence (norm)
Time (hours)
5-ph-IAA
Control (13 neurons)
Treated (17 neurons)
1.2
1
0.8
0.6
0.4
0.2
0
-2
-1
0
1
2
3
4
5
6

Supplemental Figure 2

Recognition of gephyrin fusion proteins by anti-gephyrin antibodies.

Neurons expressing mAID:mTurq2:Gephyrin or GephyrinA29:mAID:HT were fixed and labeled against gephyrin using an anti-gephyrin that recognizes the brain specific 93 kDa splice variant of gephyrin phosphorylated at Ser-270 (Synaptic Systems **#** 147 011). **A)** An example of a neuron expressing mAID:mTurq2:Gephyrin. Note that many mAID:mTurq2:Gephyrin puncta were not recognized by this antibody (some examples are shown by green arrows). **B)** An example of a neuron expressing GephyrinA29:mAID:HT labeled with the HaloTag ligand JF552-HT. Correspondence with antibody labeling is nearly perfect. Bar, 10 µm.

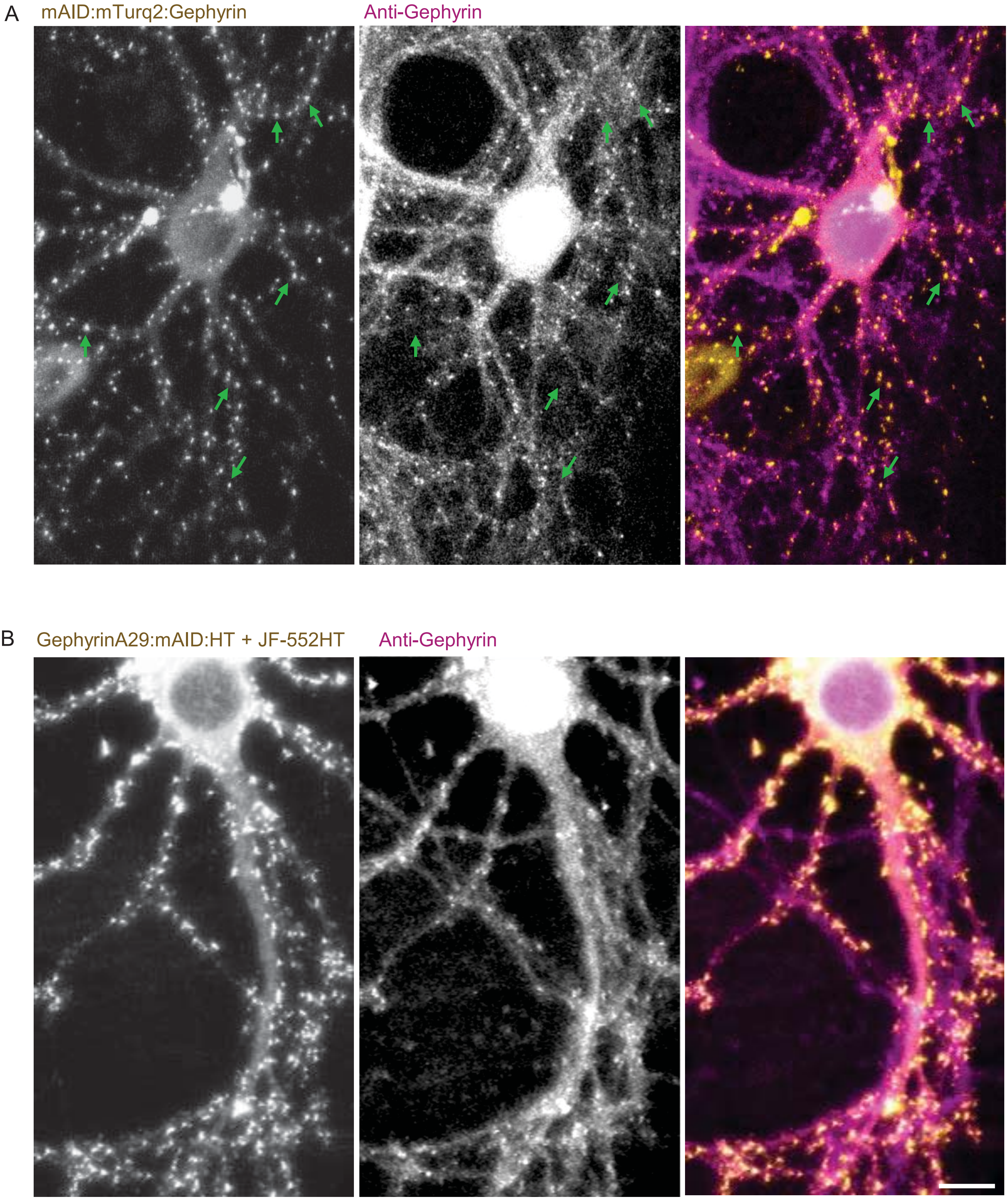
A
mAID:mTurq2:Gephyrin
Anti-Gephyrin
B
GephyrinA29:mAID:HT + JF-552HT
Anti-Gephyrin

Supplemental Figure 3:

Spatial patterns of fusion protein degradation.

A-C) Examples of neurons expressing PSD-95:mTurq2:mAID, mAID:mTurq2:GKAP and mAID:mTurq2:Gephyrin, respectively. Blue rectangles depict areas from which measurements of somatic fluorescence were obtained. Red rectangles show sites from which fluorescence measurements were made for postsynaptic proximal sites. Green rectangles show sites from which fluorescence measurements were made for postsynaptic distal sites. Scale bars: 20 µm. **D-F)** Fusion protein fluorescence measurements made at somata, proximal and distal synaptic sites of nine neurons. Each column shows data for neurons expressing the fusion protein shown in A-C. Top row shows data for the specific neurons shown in A-C), middle and bottom rows show data for additional neurons in each category. Red and green plots are averages of all tracked synapses of each neuron and category.

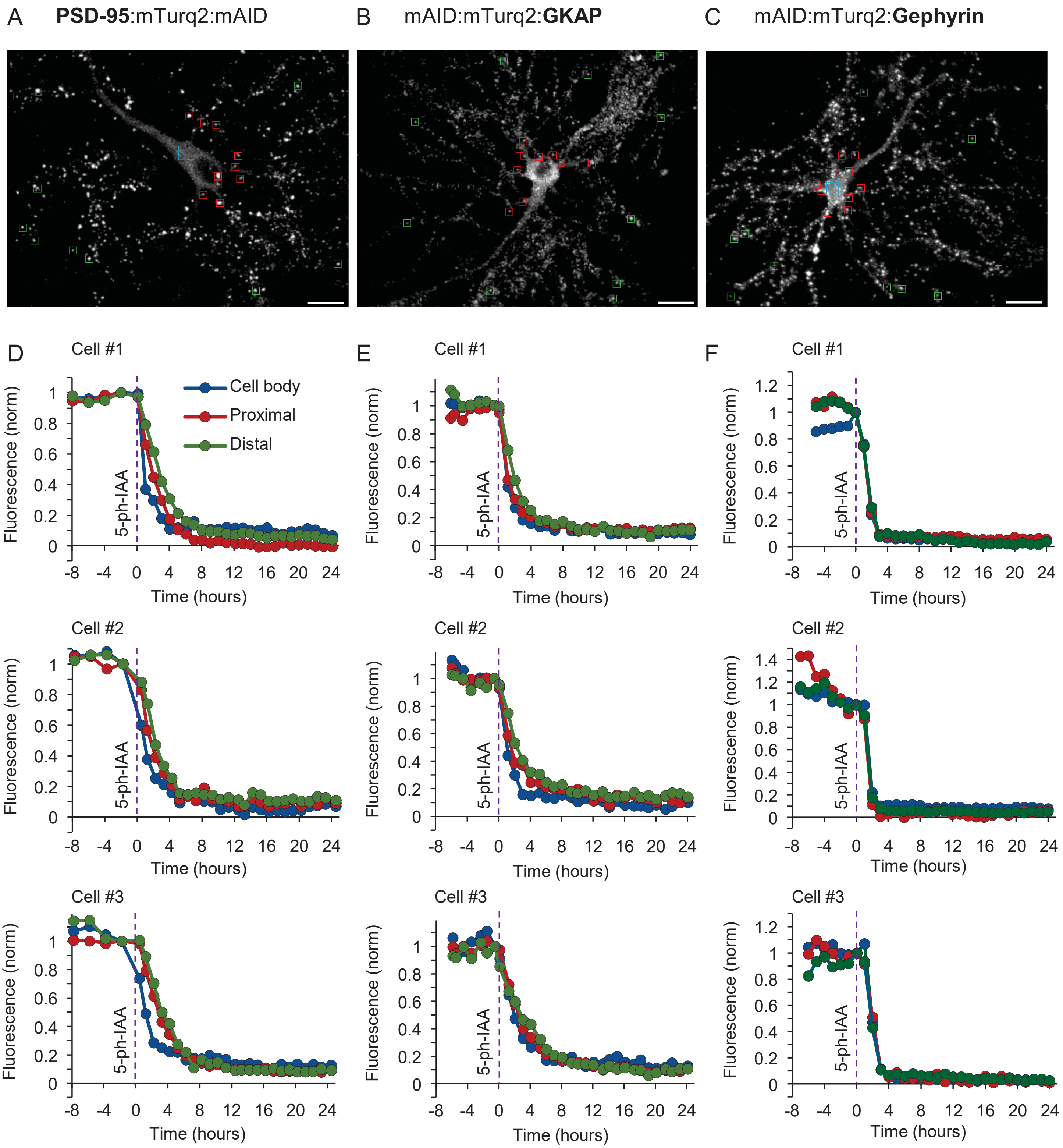
A
PSD-95:mTurq2:mAID
B
mAID:mTurq2:GKAP
C
mAID:mTurq2:Gephyrin
D
E
F
Cell #1
Cell #2
Cell #3
Cell body
Proximal
Distal
Fluorescence (norm)
Time (hours)
5-ph-IAA

Supplemental Figure 4

PSD-95:mTurq2:mAID loss rate dependencies.

A) Rates of synaptic PSD-95:mTurq2:mAID fluorescence loss (in fluorescence units / h) were calculated for each neuron coexpressing PSD-95:mTurq2:mAID and OsTIR1-P2A-mCherry by a fitting a line to mTurq2 fluorescence measurements made during the first 5 time points following exposure to 5-Ph-IAA (Inset). Loss rates were then plotted against initial PSD-95:mTurq2:mAID levels in the same neurons. The correlation between these two measures was -0.747 (34 neurons from 5 experiments). **B)** Similar plots comparing mTurq2 loss rate to mCherry fluorescence measured for the same neuron, with mCherry fluorescence serving as a surrogate for OsTIR1 expression. The correlation between these two measures was -0.651 (27 neurons from 4 experiments).

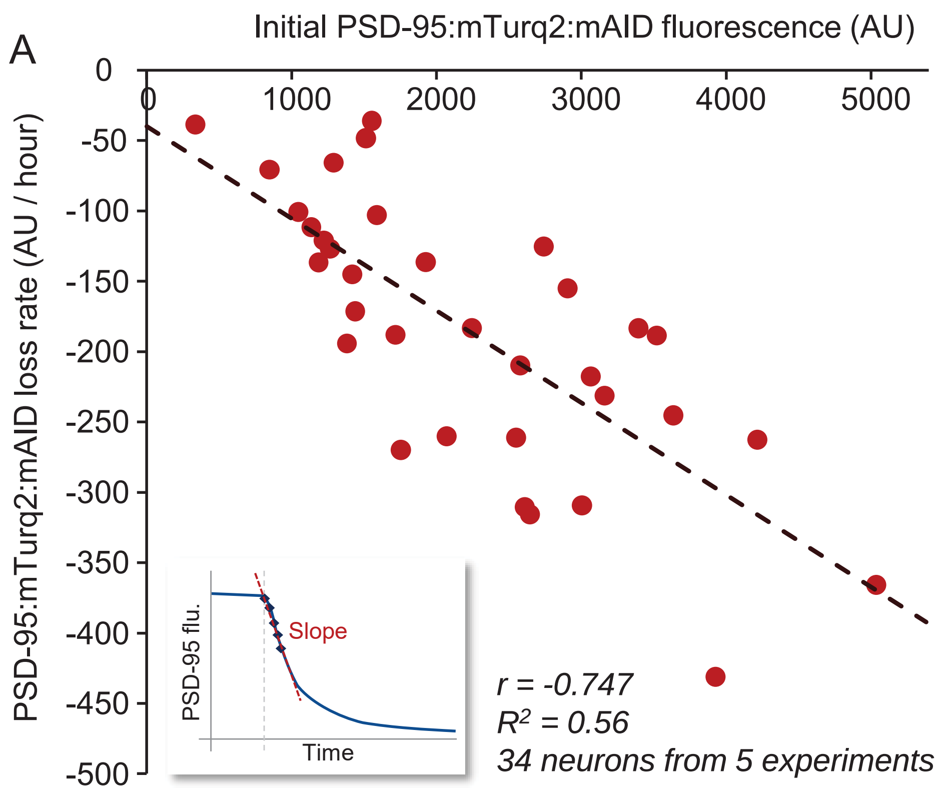
A
Initial PSD-95:mTurq2:mAID fluorescence (AU)
0
1000
2000
3000
4000
5000
PSD-95:mTurq2:mAID loss rate (AU / hour)
0
-50
-100
-150
-200
-250
-300
-350
-400
-450
-500
PSD-95 flu.
Slope
Time
r = -0.747
R2 = 0.56
34 neurons from 5 experiments

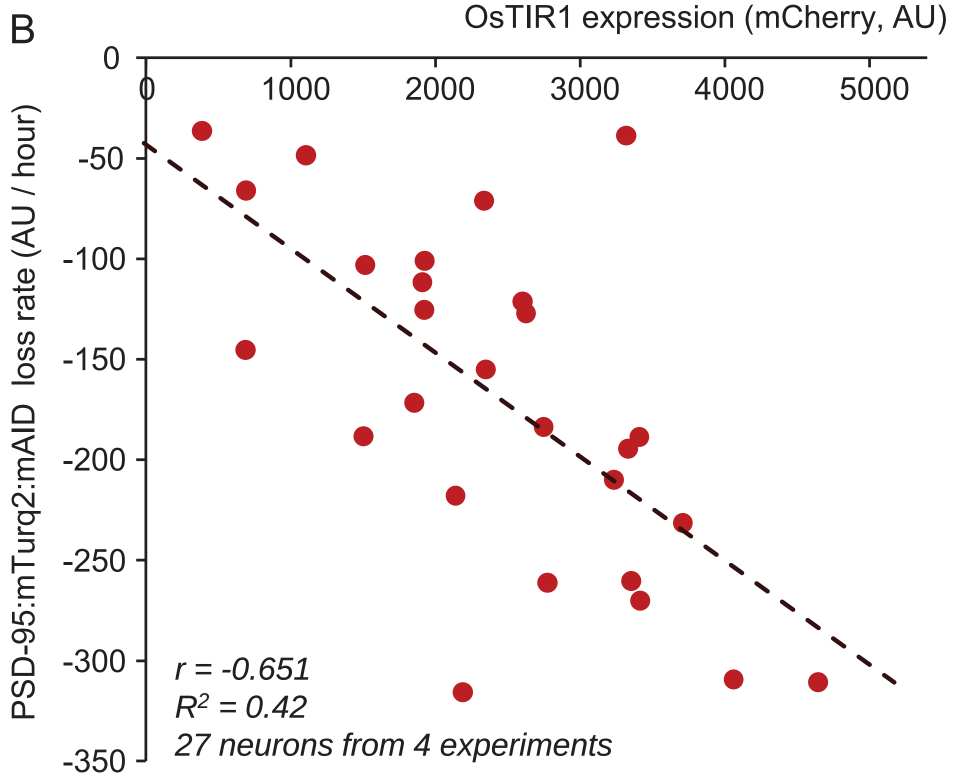
B
OsTIR1 expression (mCherry, AU)
0
1000
2000
3000
4000
5000
PSD-95:mTurq2:mAID loss rate (AU / hour)
0
-50
-100
-150
-200
-250
-300
-350
r = -0.651
R2 = 0.42
27 neurons from 4 experiments

Supplemental Figure 5

Acute degradation of PSD-95:mTurq2:mAID and GephyrinA29:mAID:HT are followed by reductions in counts of SEpH:GluA2 and SEpH:GABA$_A$Rα$_2$ puncta.

A) Changes in counts of SEpH:GluA2 puncta following exposure to 5-Ph-IAA in neurons expressing PSD-95:mTurq2:mAID and OsTIR1-P2A-mCherry. Puncta were detected automatically using a puncta detection algorithm (see Methods). Puncta counts were normalized to counts for each neuron at the last time point before exposure to 5-Ph-IAA. Data from 15 neurons from 3 separate experiments. Thick line is the population average. **B)** Changes in counts of SEpH:GluA2 puncta following exposure to 5-Ph-IAA in neurons negative for PSD-95:mTurq2:mAID. Data from 11 neurons from 3 separate experiments. Thick line is the population average. **C)** Pooled data. **D) - F)** As in A-C but for GephyrinA29:mAID:HT and SEpH:GABA$_A$Rα$_2$. All error bars are standard deviations, not SEM. Tests for difference between PSD-95:mTurq2:mAID positive and negative cells – unpaired t-tests, without assuming equal variances. * = $p<0.05$; **= $p<0.005$.

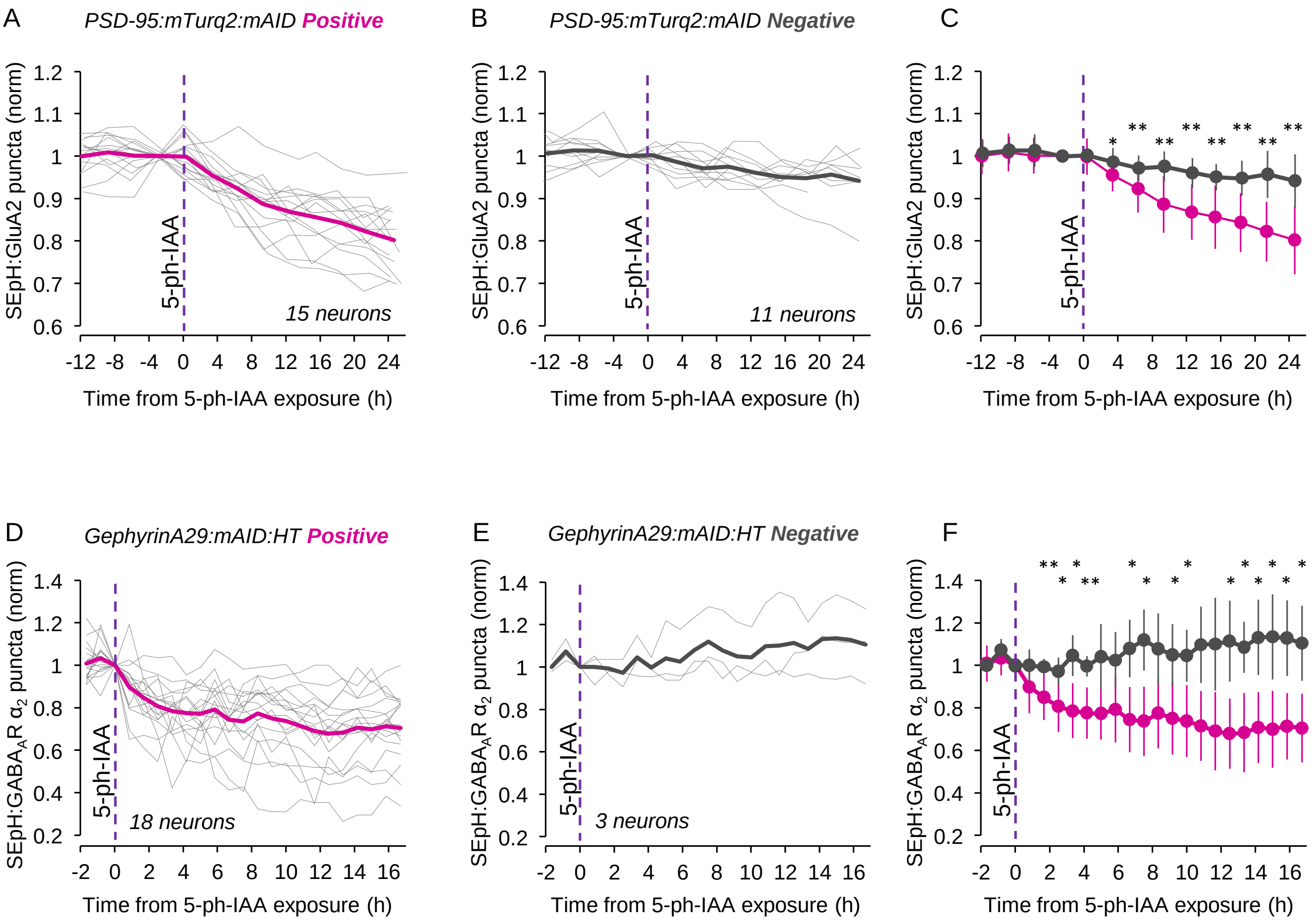

A
PSD-95:mTurq2:mAID Positive
B
PSD-95:mTurq2:mAID Negative
C
SEpH:GluA2 puncta (norm)
5-ph-IAA
15 neurons
11 neurons
Time from 5-ph-IAA exposure (h)
D
GephyrinA29:mAID:HT Positive
E
GephyrinA29:mAID:HT Negative
F
SEpH:GABA$_A$R α$_2$ puncta (norm)
18 neurons
3 neurons

Supplemental Figure 6

Quantification of PSD-95 overexpression.

Networks of neurons expressing PSD-95:mTurq2:mAID, SEpH:GluA2 and OsTIR1-P2A-mCherry as well as age matched naïve networks from the same cell culture preparations were fixed and stained against PSD-95 (FluoTag®-X2 anti-PSD95 Alexa 647; NanoTag Biotechnologies #N3702-AF647-L; 1:200). **A)** Naïve (uninfected neurons) fixed and immunolabeled against PSD-95. **B)** A neuron expressing PSD-95:mTurq2:mAID fixed and immunolabeled against PSD-95. **C)** PSD-95:mTurq2:mAID fluorescence of the same synapses as in B. Note that in this particular field of view, all synapses were PSD-95:mTurq2:mAID positive. **D)** Comparison of anti-PSD-95 immunofluorescence of postsynaptic sites positive for PSD95:mTurq:mAID in the triple infected preparations to that of postsynaptic densities in the naïve ones (15,839 and 53,913 synapses from 24 and 31 fields of view, respectively; two separate experiments, shown separately). Every data point is the average fluorescence (arbitrary units) for one field of view. Columns and error bars show means and standard deviations. Correlations (Pearson's) between PSD-95:mTurq2:mAID and anti-PSD-95 fluorescence on a synapse by synapses basis were 0.86 and 0.80 for the two experiments, indicating that anti-PSD-95 fluorescence provided a good readout of PSD-95 expression levels.

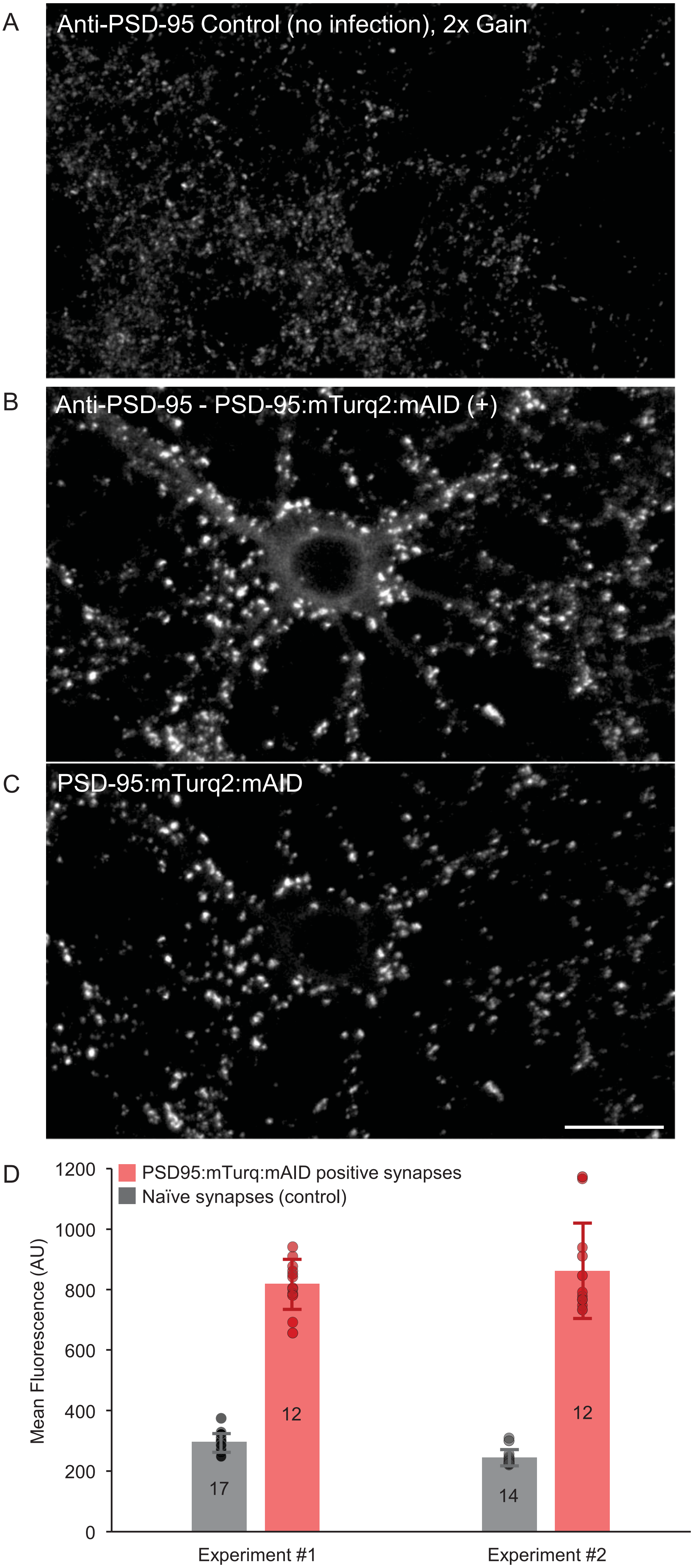

A
Anti-PSD-95 Control (no infection), 2x Gain
B
Anti-PSD-95 - PSD-95:mTurq2:mAID (+)
C
PSD-95:mTurq2:mAID
D
PSD95:mTurq:mAID positive synapses
Naïve synapses (control)
Mean Fluorescence (AU)
1200
1000
800
600
400
200
0
17
12
14
12
Experiment #1
Experiment #2

Supplemental Figure 7

Quantification of total residual synaptic PSD-95 (endogenous and exogenous) following near complete PSD-95:mTurq2:mAID degradation.

A) Illustration of experiment timeline. Stars depict imaging time points. Green, filled-in stars represent time points for images shown in B. **B)** Networks of neurons expressing PSD-95:mTurq2:mAID, SEpH:GluA2 and OsTIR1-P2A-mCherry were mounted, perfused and imaged at 6 hour intervals. Baseline images were obtained and then networks were exposed to 5-Ph-IAA (200nM). 24h later, the networks were fixed and stained against PSD-95 (see Supplemental Fig. 6 for details). Then, SEpH:GluA2 puncta were identified programmatically and ROIs were centered on these. Finally, anti PSD-95 immunofluorescence was quantified at each ROI. Age matched naïve (uninfected) neurons were also fixed and immunolabeled in identical fashion (right column). In these preparations, puncta of immunolabeled PSD-95 were identified programmatically and ROIs were centered on these, followed by PSD-95 immunofluorescence quantification in each ROI. Bar, 20 µm. **C)** PSD-95 immunofluorescence in neurons expressing PSD-95:mTurq2:mAID, SEpH:GluA2, OsTIR1-P2A-mCherry, 24 hours after exposure to 5-Ph-IAA, compared to PSD-95 immunofluorescence in naïve cells. Each circle is the average immunofluorescence of all ROIs of one field of view. Vertical bars are averages for all fields of view of one experiment. Three experiments from different cell culture preparations are shown separately. P values above data are for unpaired t-tests without assuming equal variances.

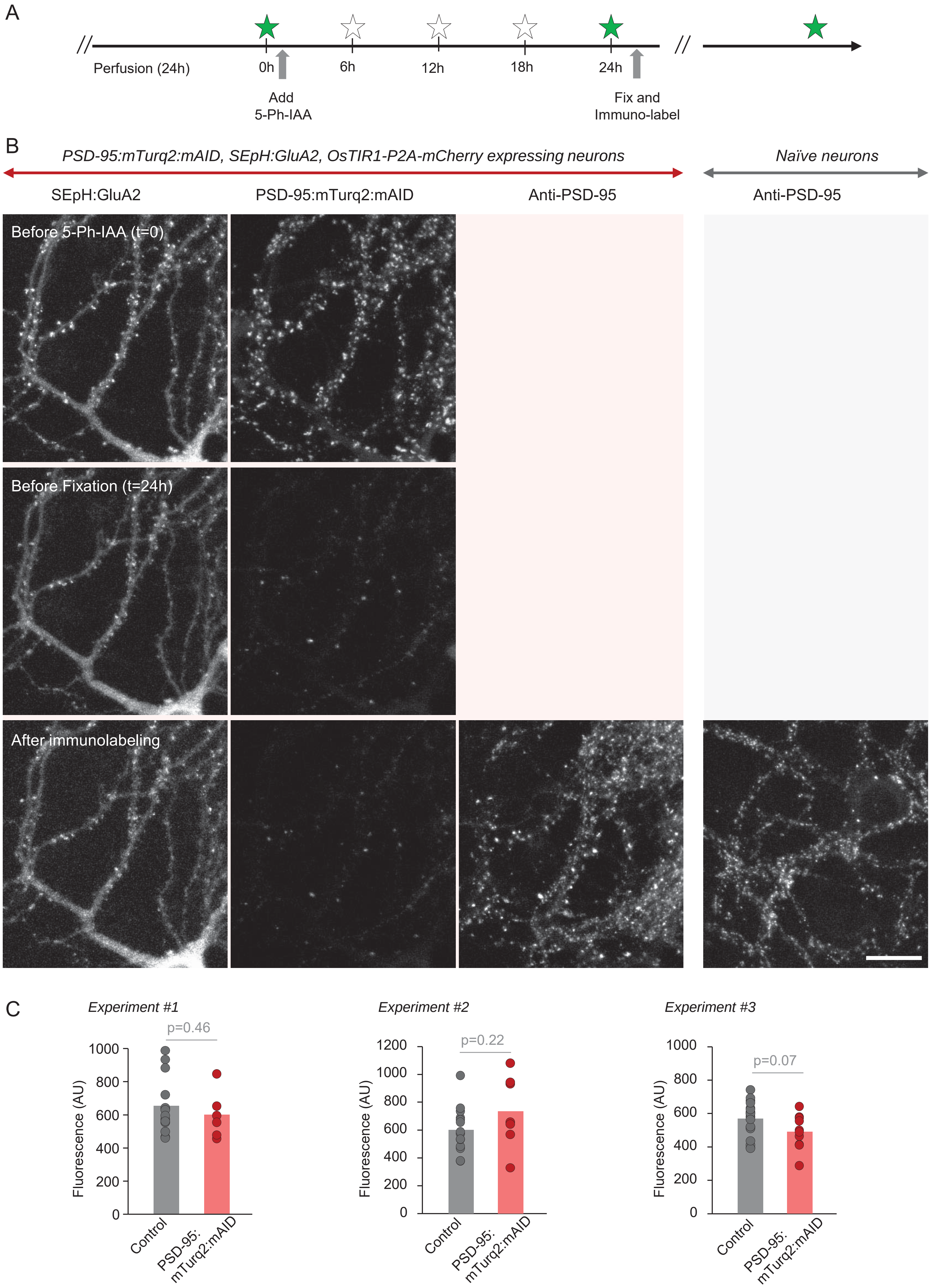

A
Perfusion (24h)
0h
6h
12h
18h
24h
Add 5-Ph-IAA
Fix and Immuno-label
B
PSD-95:mTurq2:mAID, SEpH:GluA2, OsTIR1-P2A-mCherry expressing neurons
Naïve neurons
SEpH:GluA2
PSD-95:mTurq2:mAID
Anti-PSD-95
Anti-PSD-95
Before 5-Ph-IAA (t=0)
Before Fixation (t=24h)
After immunolabeling
C
Experiment #1
p=0.46
Experiment #2
p=0.22
Experiment #3
p=0.07
Fluorescence (AU)
Control
PSD-95: mTurq2:mAID

Supplemental Figure 8

Change in protein contents of individual synapses.

A) Comparisons, on a synapse by synapse basis, of changes in SEpH:GluA2 fluorescence as a function of PSD-95:mTurq2:mAID fluorescence loss at the same synapses measured 15 hours after exposure to 5-Ph-IAA. 455 synapses from 18 neurons in 3 separate experiments. Dashed line is a linear regression for these data, with its equation shown below. r = Pearson's correlation. **B)** Dependence of changes in SEpH:GluA2 fluorescence as a function of initial SEpH:GluA2 fluorescence at the same synapses. Dashed line is a linear regression for these data, with its equation shown below. The scatter around the line, quantified by the coefficient of determination (R^2) is 0.309. Orange line and right hand Y axis show the distribution of initial SEpH:GluA2 fluorescence values for these 455 synapses. **C)** Dependence of changes in PSD-95:mTurq2:mAID fluorescence as a function of initial PSD-95:mTurq2:mAID fluorescence at the same synapses. Dashed line is a linear regression for these data, with its equation shown below. Orange line and right hand Y axis shows the distribution of initial PSD-95:mTurq2:mAID fluorescence values for these 455 synapses. Note the absence of any synapse for which PSD-95:mTurq2:mAID fluorescence increased following 5-Ph-IAA exposure. Also note the tighter correlation and reduced scatter (R^2 = 0.876) for these data as compared to B. Limiting the regression analysis to synapses with fluorescence levels comparable to those of SEpH:GluA2 in panel B (PSD-95:mTurq2:mAID no brighter than 2,000 fluorescence units and Δ fluorescence no greater that 800 units; gray rectangle; 271 synapses) resulted in a correlation of r = 0.826 (R^2 = 0.682). The reduced scatter and tighter correlation for fluorescent objects of similar fluorescence levels indicates that the moderate dependence of SEpH:GluA2 loss on PSD-95:mTurq2:mAID (panel A), including observations of increased SEpH:GluA2 fluorescence, cannot be solely attributed to measurement noise. **D)** Distribution of ratios of SEpH:GluA2 fluorescence loss to PSD-95:mTurq2:mAID fluorescence loss at individual synapses. Negative values reflect gains of SEpH:GluA2 fluorescence. **E)** Comparisons, on a synapse by synapse basis, of changes in SEpH:$GABA_AR\alpha_2$ fluorescence as a function of GephyrinA29:mAID:HT fluorescence loss at the same synapses. 900 synapses from 18 neurons in 3 separate experiments. Dashed line is a linear regression for these data, with its equation is shown below. **F)** Comparisons, on a synapse by synapse basis, of changes in PSD-95:mCit fluorescence as a function of mAID:mTurq2:GKAP fluorescence loss at the same synapses 15 hours after exposure to 5-Ph-IAA. 1,723 synapses from 39 neurons in 5 separate experiments. Dashed line is a linear regression for these data, with its equation is shown below. All fluorescence data in this figure reflect averages of measurements made at three consecutive time points to minimize effects of measurement noise.

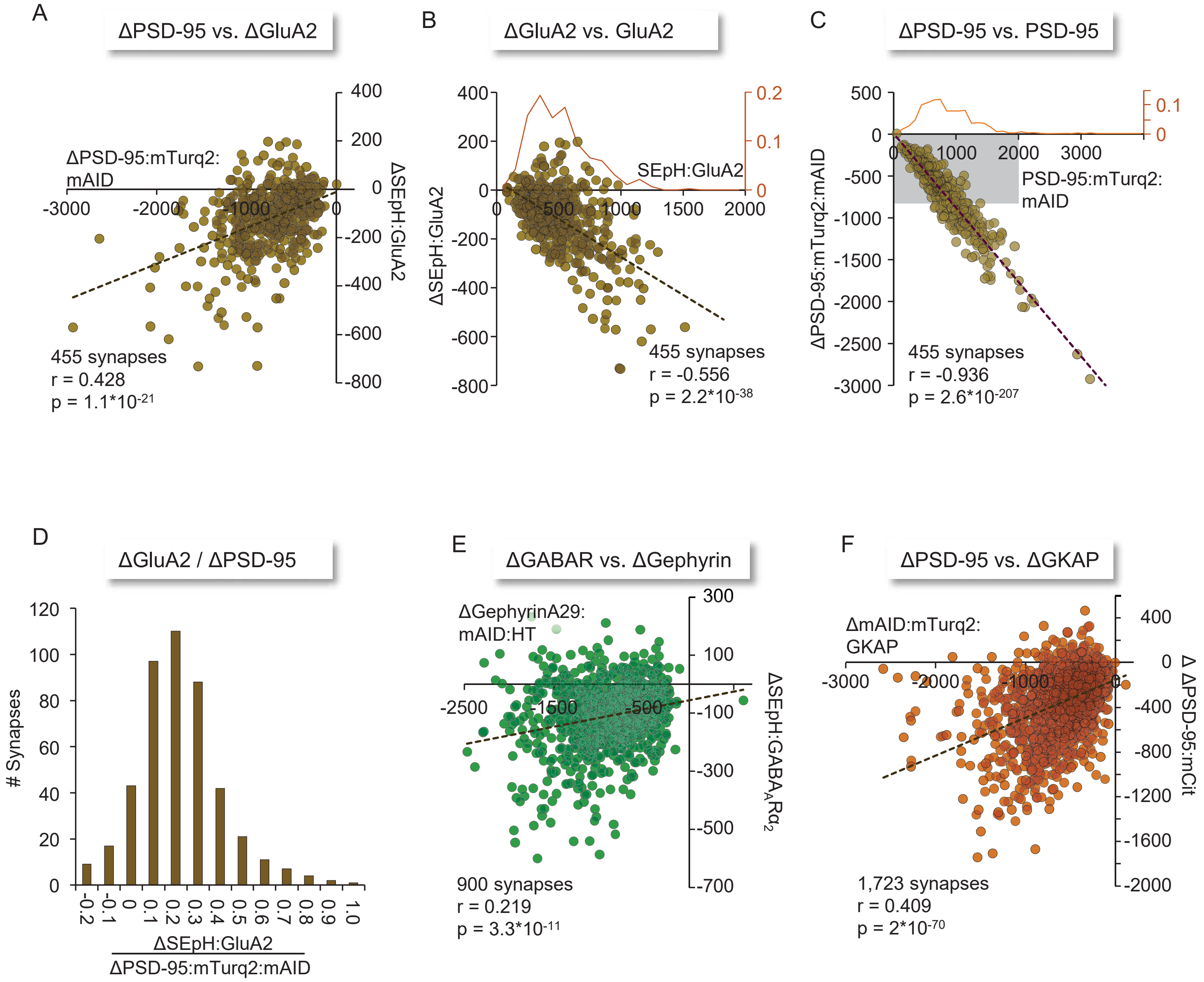

A
ΔPSD-95 vs. ΔGluA2
ΔPSD-95:mTurq2:
mAID
ΔSEpH:GluA2
455 synapses
r = 0.428
p = 1.1*10-21
B
ΔGluA2 vs. GluA2
SEpH:GluA2
ΔSEpH:GluA2
455 synapses
r = -0.556
p = 2.2*10-38
C
ΔPSD-95 vs. PSD-95
PSD-95:mTurq2:
mAID
ΔPSD-95:mTurq2:mAID
455 synapses
r = -0.936
p = 2.6*10-207
D
ΔGluA2 / ΔPSD-95
Synapses
ΔSEpH:GluA2
ΔPSD-95:mTurq2:mAID
E
ΔGABAR vs. ΔGephyrin
ΔGephyrinA29:
mAID:HT
ΔSEpH:GABAARα2
900 synapses
r = 0.219
p = 3.3*10-11
F
ΔPSD-95 vs. ΔGKAP
ΔmAID:mTurq2:
GKAP
Δ ΔPSD-95:mCit
1,723 synapses
r = 0.409
p = 2*10-70

Supplemental Figure 9

Quantification of Gephyrin overexpression.

Preparations containing neurons expressing GephyrinA29:mAID:HT, SEpH:GABA$_A$Rα_2 and OsTIR1-P2A-mCherry as well as age matched naïve networks from the same cell culture preparations were fixed and stained against Gephyrin (monoclonal mouse anti gephyrin Synaptic Systems #147111). Infected networks were labeled before fixation with JF552-HT. **A)** Naïve (uninfected neurons) fixed and immunolabeled against gephyrin. **B)** A neuron expressing GephyrinA29:mAID:HT fixed and immunolabeled against gephyrin. **C)** GephyrinA29:mAID:HT + JF552-HT fluorescence of the same synapses as in B. **D)** Comparison of Anti-Gephyrin immunofluorescence of postsynaptic sites positive to JF552-HT fluorescence in the triple infected networks to that of postsynaptic densities in the naïve networks (6,204 and 4,626 synapses from 16 and 16 fields of view, respectively; two separate experiments, shown separately). Every data point is the average fluorescence (arbitrary units) for one field of view. Columns and error bars show means and standard deviations. Correlation (Pearson's) between JF552-HT fluorescence and anti-Gephyrin fluorescence on a synapse by synapses basis was 0.50, indicating that anti-Gephyrin fluorescence provided a reasonable, if imperfect, readout of gephyrin expression levels, possibly affected by the use of a HT - HT ligand pair rather than a fluorescent protein

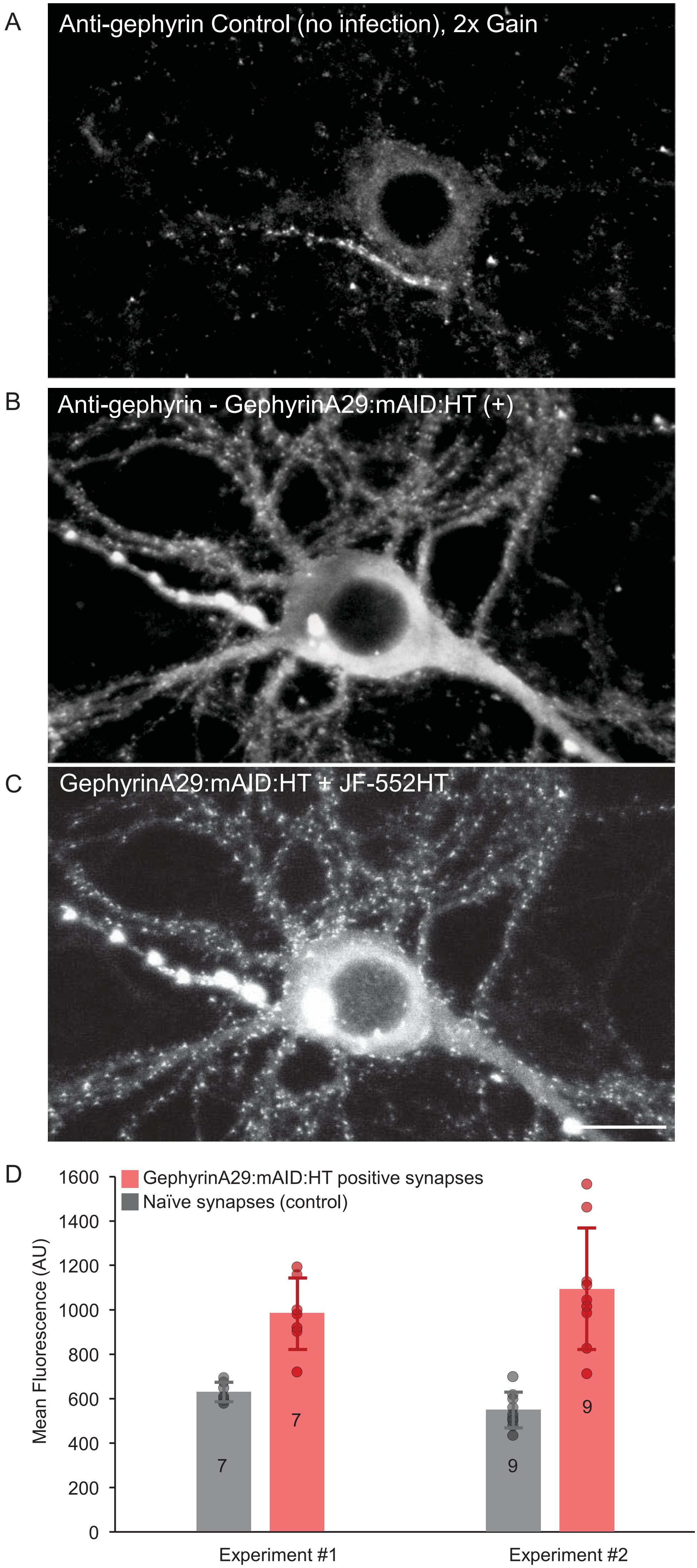
A
Anti-gephyrin Control (no infection), 2x Gain
B
Anti-gephyrin - GephyrinA29:mAID:HT (+)
C
GephyrinA29:mAID:HT + JF-552HT
D
GephyrinA29:mAID:HT positive synapses
Naïve synapses (control)
Mean Fluorescence (AU)
1600
1400
1200
1000
800
600
400
200
0
7
7
9
9
Experiment #1
Experiment #2

Supplemental Figure 10

Quantification of total residual synaptic Gephyrin (endogenous and exogenous) following near complete GephyrinA29:mAID:HT degradation.

A) Illustration of experiment timeline. Stars depict imaging time points. Green, filled-in stars represent time points for images shown in B. **B)** Networks of neurons expressing GephyrinA29:mAID:HT (labeled with JF635-HT) , SEpH:GABA$_A$Rα_2 and OsTIR1-P2A-mCherry were mounted, perfused and imaged at 6 hour intervals. Baseline images were obtained and then networks were exposed to 5-Ph-IAA (200nM). 12h later, the networks were fixed and stained against Gephyrin (see Supplemental Fig. 9 for details). Then, SEpH:GABA$_A$Rα_2 puncta were identified programmatically and ROIs were centered on these. Finally, anti-Gephyrin immunofluorescence was quantified at each ROI. Age matched naïve (uninfected) neurons were also fixed and immunolabeled in identical fashion (right column). In these preparations, puncta of immunolabeled Gephyrin were identified programmatically and ROIs were centered on these, followed by Gephyrin immunofluorescence quantification in each ROI. Bar, 20 µm. **C)** Gephyrin immunofluorescence in neurons expressing GephyrinA29:mAID:HT, SEpH:GABA$_A$Rα_2 and OsTIR1-P2A-mCherry, 24 hours after exposure to 5-Ph-IAA, compared to Gephyrin immunofluorescence in naïve cells. Each circle is the average immunofluorescence of all ROIs of one field of view. Vertical bars are averages for all fields of view of one experiment. Three experiments from different cell culture preparations are shown separately. P values above data are for unpaired t-tests without assuming equal variances.

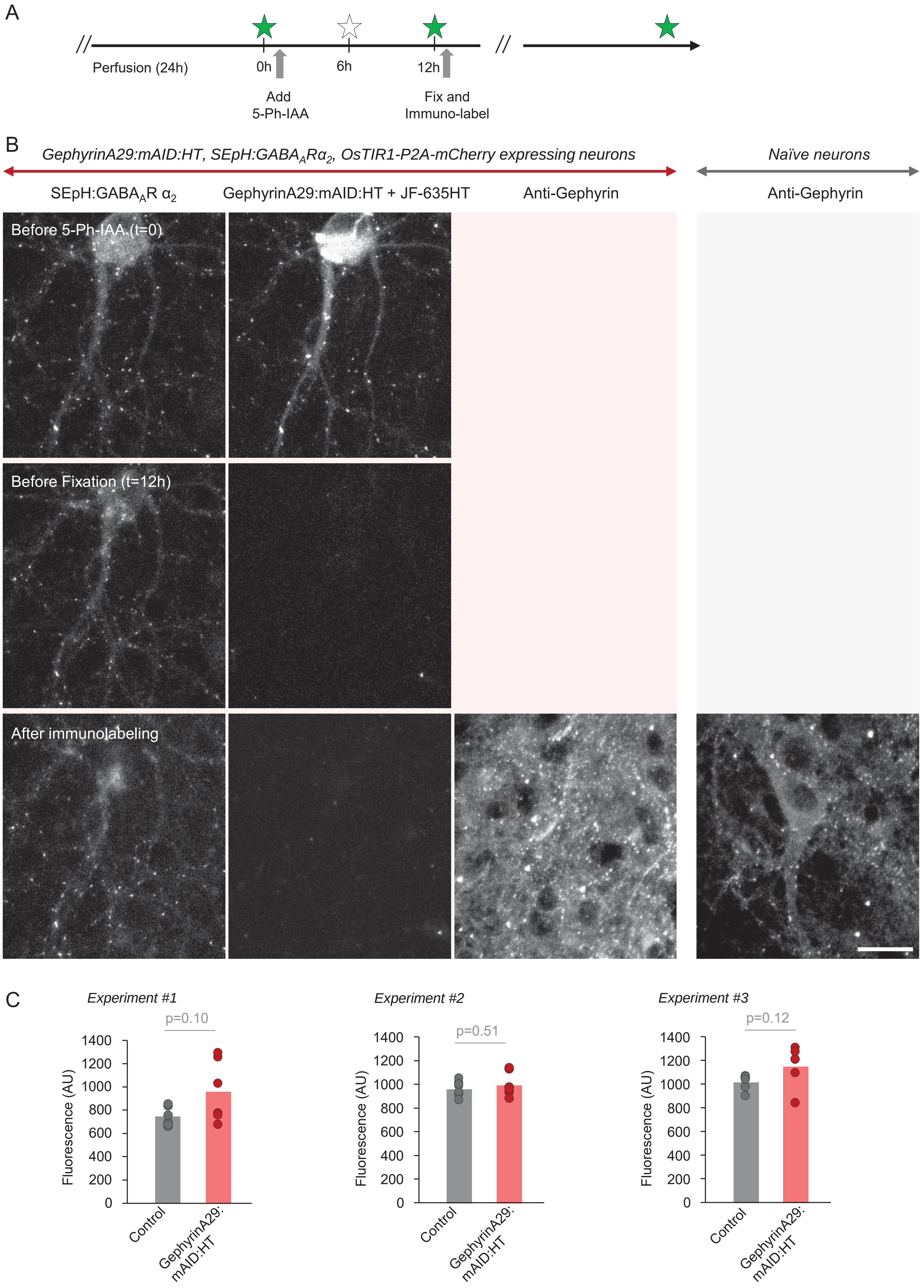

A
Perfusion (24h)
0h
6h
12h
Add
5-Ph-IAA
Fix and
Immuno-label
B
GephyrinA29:mAID:HT, SEpH:GABA$_A$Rα$_2$, OsTIR1-P2A-mCherry expressing neurons
Naïve neurons
SEpH:GABA$_A$R α$_2$
GephyrinA29:mAID:HT + JF-635HT
Anti-Gephyrin
Anti-Gephyrin
Before 5-Ph-IAA (t=0)
Before Fixation (t=12h)
After immunolabeling
C
Experiment #1
p=0.10
Experiment #2
p=0.51
Experiment #3
p=0.12
Fluorescence (AU)
1400
1200
1000
800
600
400
200
0
Control
GephyrinA29:
mAID:HT

Supplemental Figure 11

Acute degradation of mAID:mTurq2:Gephyrin is followed by loss of GABA receptors at the same synapses.

A) Top panels: A rat cortical neuron in culture co-expressing mAID:mTurq2:Gephyrin, SEpH:$GABA_AR\alpha_2$ and OsTIR1-P2A-mCherry (not shown). Bottom panels: Region in yellow rectangle at greater detail, before, and after addition of 5-Ph-IAA. Note that the near complete loss of fluorescence (presumably reflecting mAID:mTurq2:Gephyrin degradation) is associated with a partial reduction in SEpH:$GABA_AR\alpha_2$ fluorescence. Scale bars: 10µm (top panels) 5µm (bottom panels). **B)** mAID:mTurq2:Gephyrin fluorescence measured at 50 synapses of each neuron tracked throughout the experiments. Each thin gray line is the average mAID:mTurq2:Gephyrin fluorescence (normalized to the time point just before 5-Ph-IAA addition) measured for the synapses of one neuron (18 neurons from 3 experiments). Thick magenta line is the population average**. C)** changes in SEpH:$GABA_AR\alpha_2$ fluorescence at the same synapses and neurons of B. Thick brown line is the population average. **D)** SEpH:$GABA_AR\alpha_2$ fluorescence measured at 200 synapses of neurons positive for SEpH:$GABA_AR\alpha_2$ and OsTIR1-P2A-mCherry but negative for mAID:mTurq2:Gephyrin. Each thin gray line is the average fluorescence measured for the synapses of one neuron (4 neurons from 1 experiment). Thick gray line is the population average. **E)** Pooled data. Error bars are standard deviations. Test for difference between mAID:mTurq2:Gephyrin positive and negative cells – unpaired t-test, without assuming equal variances; applied to data from last time point.

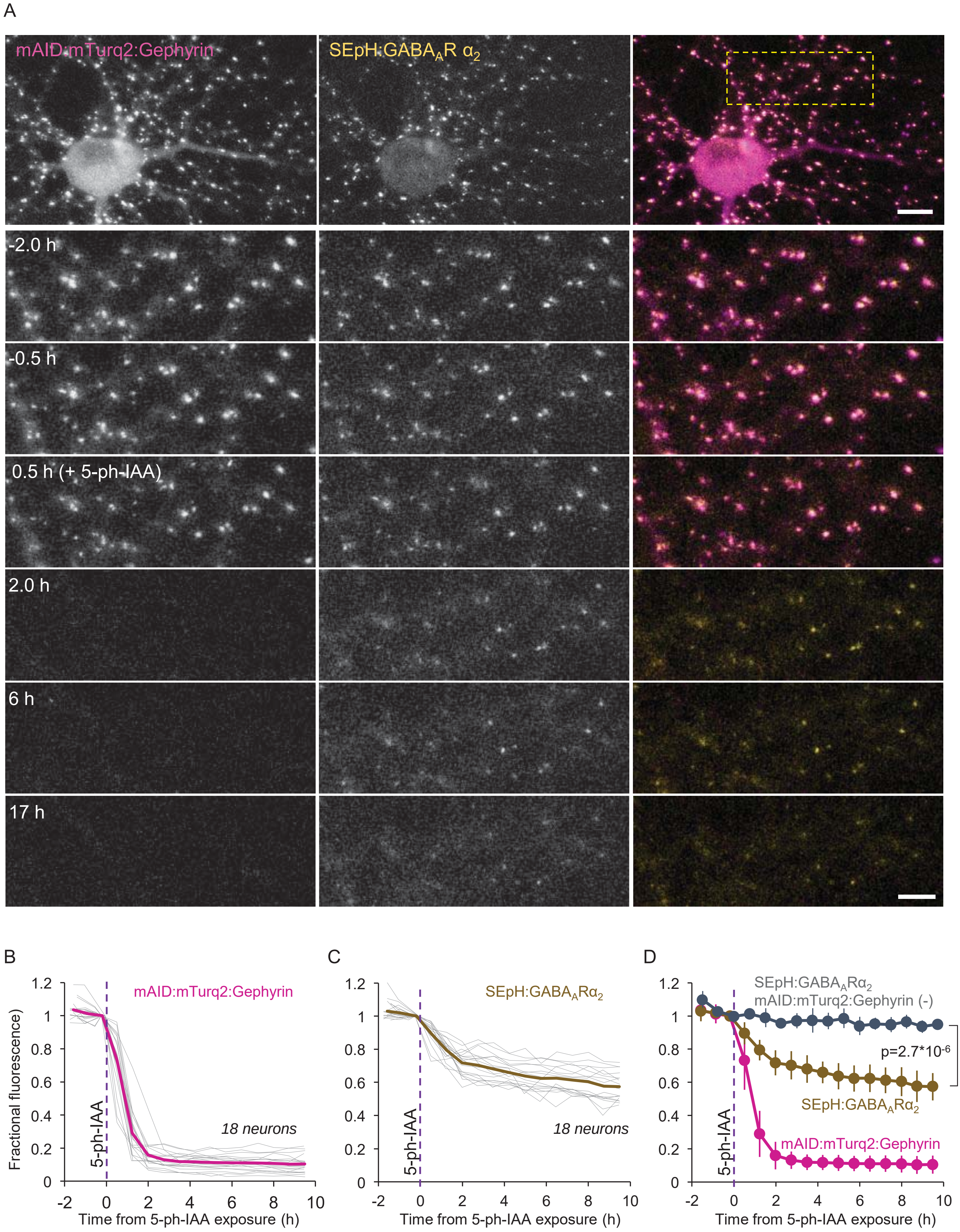
A
mAID:mTurq2:Gephyrin
SEpH:GABA$_A$R α_2
-2.0 h
-0.5 h
0.5 h (+ 5-ph-IAA)
2.0 h
6 h
17 h
B
mAID:mTurq2:Gephyrin
Fractional fluorescence)
5-ph-IAA
18 neurons
Time from 5-ph-IAA exposure (h)
C
SEpH:GABA$_A$Rα_2
5-ph-IAA
18 neurons
Time from 5-ph-IAA exposure (h)
D
SEpH:GABA$_A$Rα_2
mAID:mTurq2:Gephyrin (-)
p=2.7*10^{-6}
SEpH:GABA$_A$Rα_2
mAID:mTurq2:Gephyrin
5-ph-IAA
Time from 5-ph-IAA exposure (h)

Supplemental Figure 12

Acute degradation of PSD-95:mTurq2:mAID is followed by GKAP influx into the same synapses.

A) Cortical neurons in culture were infected with lentiviral expression vectors encoding for PSD-95:mTurq2:mAID, mCit:GKAP and OsTIR1-P2A-mCherry. Changes in mCit:GKAP fluorescence were measured at synapses in fields of view containing neurons expressing all three exogenous proteins. Here, unlike Fig. 7, synapses were not tracked individually but located (segmented) programmatically anew at each time step, resulting in an unbiased selection of synapses but with no distinction between synapses belonging to triple expressing neurons and synapses belonging to other neurons in the same fields of view. Each thin gray line is the average fluorescence measured for the synapses in one field of view (13 fields of view from 3 experiments). Thick brown line is the population average. **B)** mCit:GKAP fluorescence measured at synapses in fields of view containing neurons positive for mCit:GKAP and OsTIR1-P2A-mCherry but negative for PSD-95:mTurq2:mAID. Each thin gray line is the average fluorescence measured for the synapses of one neuron (9 neurons from 3 experiments). Thick gray line is the population average. **C)** PSD-95:mTurq2:mAID fluorescence measured at the same synapses and neurons of A. Thick magenta line is the population average. **D)** Pooled data. Error bars are standard deviations. Test for difference between PSD-95:mTurq2:mAID positive and negative cells – unpaired t-test, without assuming equal variances; applied to data from t=30 h.

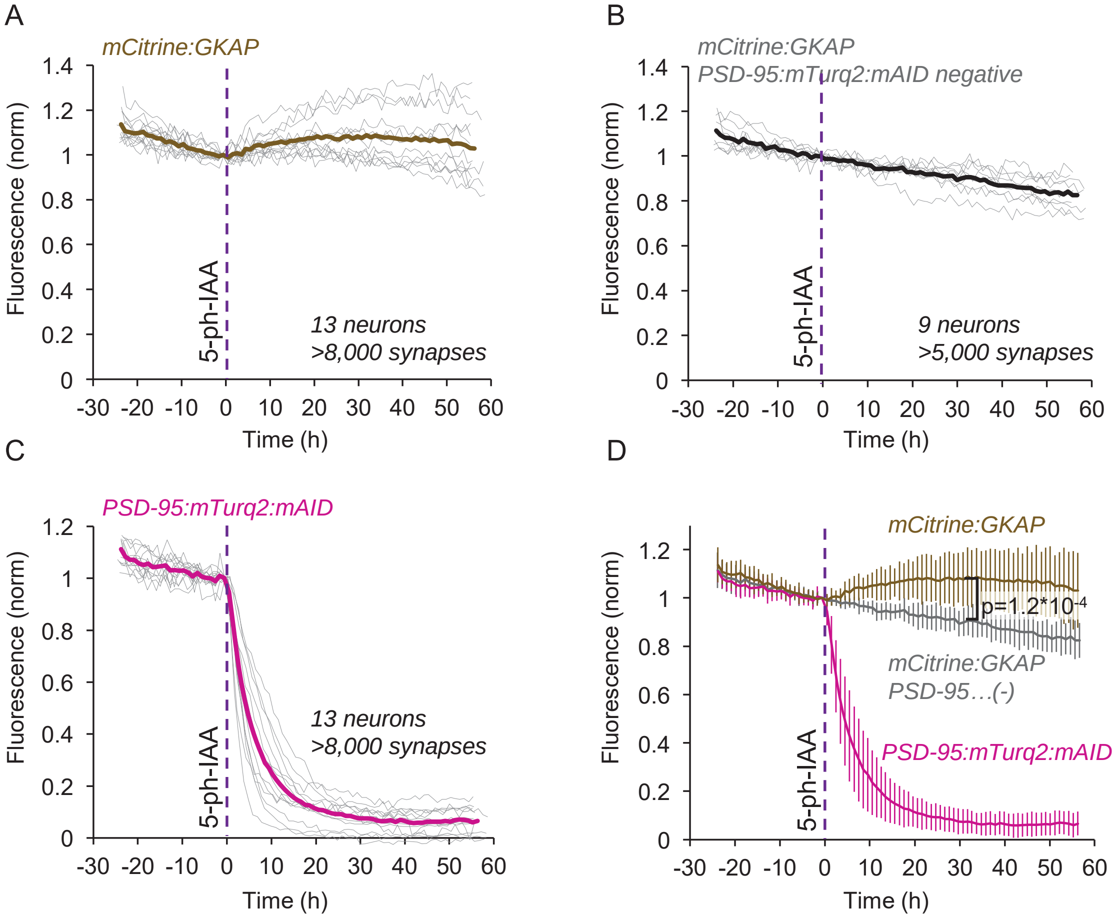
A
mCitrine:GKAP
Fluorescence (norm)
5-ph-IAA
13 neurons
>8,000 synapses
Time (h)
B
mCitrine:GKAP
PSD-95:mTurq2:mAID negative
Fluorescence (norm)
5-ph-IAA
9 neurons
>5,000 synapses
Time (h)
C
PSD-95:mTurq2:mAID
Fluorescence (norm)
5-ph-IAA
13 neurons
>8,000 synapses
Time (h)
D
mCitrine:GKAP
p=1.2*10-4
mCitrine:GKAP
PSD-95…(-)
PSD-95:mTurq2:mAID
Fluorescence (norm)
5-ph-IAA
Time (h)

Supplemental Figure 13

Acute degradation of PSD-95:mTurq2:mAID is followed by rapid replacement with PSD-95:mCitrine.

A) A rat cortical neuron in culture co-expressing PSD-95:mTurq2:mAID (left) and PSD-95:mCitrine (middle) as well as OsTIR1-P2A-mCherry (not shown). **B)** 5-Ph-IAA induced nearly complete loss of PSD-95:mTurq2:mAID, and increased synaptic levels of PSD-95:mCitrine at the same synapses. Scale bar: 20 µm. **C)** Changes in PSD-95:mCitrine fluorescence measured at 15-34 synapses of each neuron tracked throughout the experiments (485 in total). Each thin gray line is the average normalized fluorescence measured for the synapses of one neuron (22 neurons from 3 experiments). Thick brown line is the population average. **D)** PSD-95:mTurq2:mAID fluorescence measured at the same synapses and neurons of C. Thick magenta line is the population average. **E)** PSD-95:mCitrine fluorescence measured at 14-42 synapses of neurons positive for PSD-95:mCitrine and OsTIR1-P2A-mCherry but negative for PSD-95:mTurq2:mAID (380 in total). Each thin gray line is the average fluorescence measured for the synapses of one neuron (14 neurons from 3 experiments). Thick gray line is the population average. **F)** Pooled data. Error bars are standard deviations. Test for difference between PSD-95:mTurq2:mAID positive and negative cells – unpaired t-test, without assuming equal variances; applied to data from last time point.

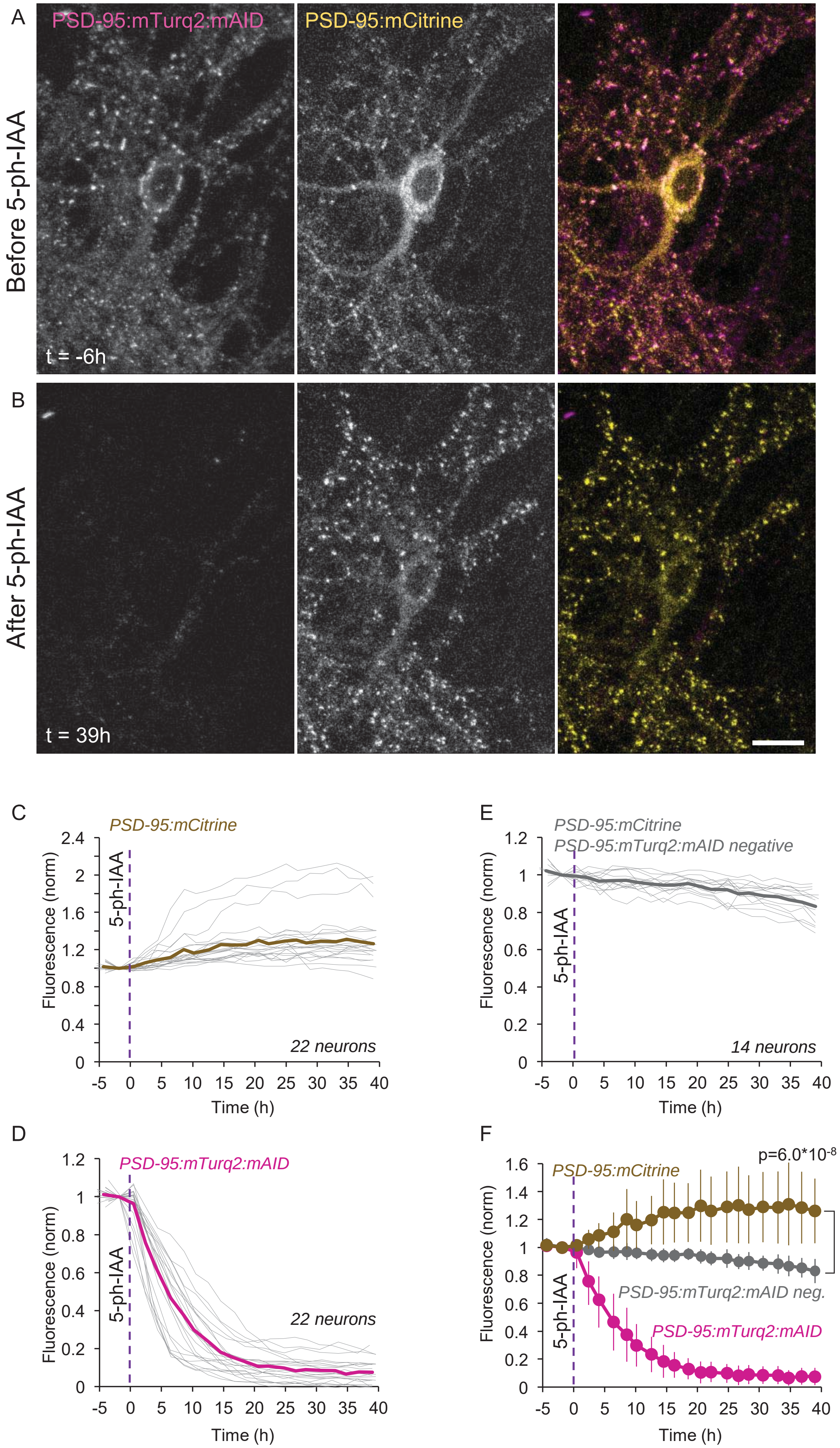
A
PSD-95:mTurq2:mAID
PSD-95:mCitrine
Before 5-ph-IAA
t = -6h
B
After 5-ph-IAA
t = 39h
C
PSD-95:mCitrine
5-ph-IAA
22 neurons
Fluorescence (norm)
Time (h)
D
PSD-95:mTurq2:mAID
5-ph-IAA
22 neurons
Fluorescence (norm)
Time (h)
E
PSD-95:mCitrine
PSD-95:mTurq2:mAID negative
5-ph-IAA
14 neurons
Fluorescence (norm)
Time (h)
F
PSD-95:mCitrine
p=6.0*10-8
PSD-95:mTurq2:mAID neg.
PSD-95:mTurq2:mAID
5-ph-IAA
Fluorescence (norm)
Time (h)

Supplemental Figure 14

Data processing and analysis of *in-vivo* experiments.

A) Processing and analysis pipeline for two-photon image stacks to quantify expression of the cytosolically expressed mAID:EGFP (Fig. 3). Single image planes (syn. fields of view, FOV) were matched and aligned across all imaging time points. Cytosolic regions of interest (ROIs) were identified as ring-shaped areas round the nuclear H2B:mCherry blobs. The single-cell EGFP signal was quantified normalizing the mean green cytosolic fluorescence by the mean nuclear red fluorescence. Only cells with a reliable baseline signal ($F_{norm} \geq 0.3$) were included in the analysis (see Methods). **B)** Analysis of synaptic PSD-95 signals (Fig. 4). For both the PSD-95:FingR:EGFP signal and the PSD-95:JF635-HT signal, neuropil fluorescence was quantified by manually selecting representative circular ROIs from the images (yellow circles). Moreover, background signals were assessed by estimating fluorescence in ROIs on blood vessels (blue circles). The bulk synaptic PSD-95 signal was then quantified as the mean background-normalized neuropil fluorescence.

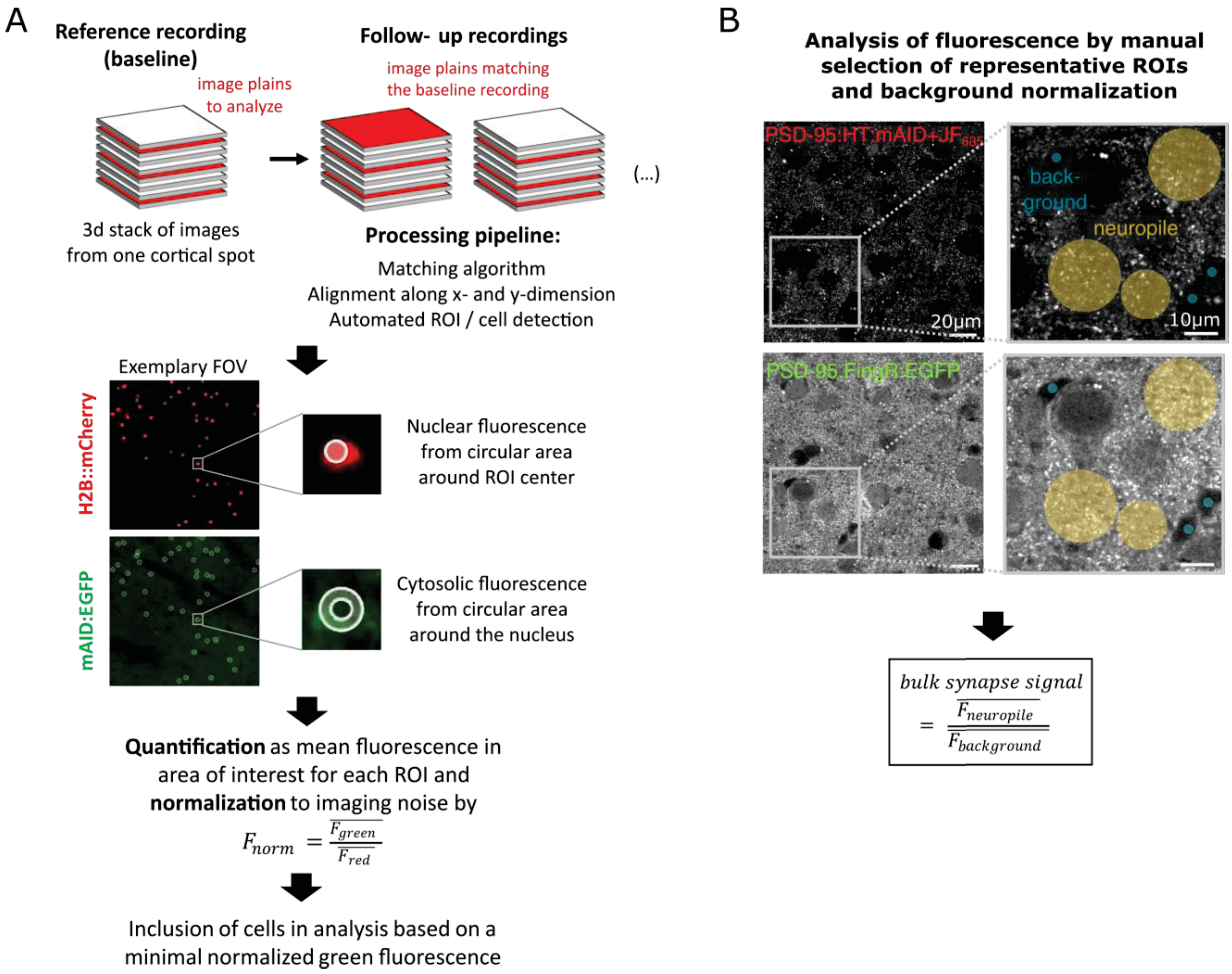
A
Reference recording (baseline)
image plains to analyze
Follow- up recordings
image plains matching the baseline recording
(...)
3d stack of images from one cortical spot
Processing pipeline:
Matching algorithm
Alignment along x- and y-dimension
Automated ROI / cell detection
Exemplary FOV
H2B::mCherry
mAID:EGFP
Nuclear fluorescence from circular area around ROI center
Cytosolic fluorescence from circular area around the nucleus
Quantification as mean fluorescence in area of interest for each ROI and normalization to imaging noise by
$F_{norm} = \frac{F_{green}}{F_{red}}$
Inclusion of cells in analysis based on a minimal normalized green fluorescence
B
Analysis of fluorescence by manual selection of representative ROIs and background normalization
PSD-95:HT:mAID+JF
back-ground
neuropile
20µm
10µm
PSD-95.FingR:EGFP
bulk synapse signal
$= \frac{F_{neuropile}}{F_{background}}$

References mentioned in Supplementary Information figure legends

Yesbolatova A, Saito Y, Kitamoto N, Makino-Itou H, Ajima R, Nakano R, Nakaoka H, Fukui K, Gamo K, Tominari Y, Takeuchi H, Saga Y, Hayashi KI, Kanemaki MT. (2020) The auxin-inducible degron 2 technology provides sharp degradation control in yeast, mammalian cells, and mice. Nat Commun. 11:5701.
