# Supplementary material for: Revealing acute consequences of rapid degradation of synaptic fusion proteins at individual synapses using Auxin-Inducible Degron 2 technology
Source: Commun Biol. 2025 Nov 17;8:1589. doi: 10.1038/s42003-025-08996-8 (PMC12623870; doi:10.1038/s42003-025-08996-8)
Supplement: Supplementary file 6 — Description of Additional Supplementary Files [file 42003_2025_8996_MOESM6_ESM.pdf]

Description of Additional Supplementary Files

File Name: Supplemental Movie 1
Description: Time lapse videos demonstrating the loss of fusion protein fluorescence of PSD-95:mTurq2:mAID, mAID:mTurq2:GKAP and mAID:mTurq2:Gephyrin. Same neurons as those shown in Supplemental Fig. 2.

File name: Supplementary Data 1
Description: Source data behind graphs in main figures concerning experiments carried out in cell culture.

File name: Supplementary Data 2
Description: Source data behind graphs in main figures concerning experiments carried out *in vivo*.

File name: Supplementary Data 3
Description: Annotated sequences of all plasmids used in study (.seq, .gb or .gbk files)
