# Supplementary material for: Revealing acute consequences of rapid degradation of synaptic fusion proteins at individual synapses using Auxin-Inducible Degron 2 technology
Source: Commun Biol. 2025 Nov 17;8:1589. doi: 10.1038/s42003-025-08996-8 (PMC12623870; doi:10.1038/s42003-025-08996-8)
Supplement: Supplementary file 7 — Reporting summary [file 42003_2025_8996_MOESM7_ESM.pdf]

nature portfolio

Corresponding author(s): Noam E Ziv

Last updated by author(s): Sep 3, 2025

Reporting Summary

Nature Portfolio wishes to improve the reproducibility of the work that we publish. This form provides structure for consistency and transparency in reporting. For further information on Nature Portfolio policies, see our Editorial Policies and the Editorial Policy Checklist.

Statistics

For all statistical analyses, confirm that the following items are present in the figure legend, table legend, main text, or Methods section.

n/a	Confirmed	
☐	☒	The exact sample size (n) for each experimental group/condition, given as a discrete number and unit of measurement
☐	☒	A statement on whether measurements were taken from distinct samples or whether the same sample was measured repeatedly
☐	☒	The statistical test(s) used AND whether they are one- or two-sided<br>*Only common tests should be described solely by name; describe more complex techniques in the Methods section.*
☐	☒	A description of all covariates tested
☐	☒	A description of any assumptions or corrections, such as tests of normality and adjustment for multiple comparisons
☐	☒	A full description of the statistical parameters including central tendency (e.g. means) or other basic estimates (e.g. regression coefficient) AND variation (e.g. standard deviation) or associated estimates of uncertainty (e.g. confidence intervals)
☒	☐	For null hypothesis testing, the test statistic (e.g. F, t, r) with confidence intervals, effect sizes, degrees of freedom and P value noted<br>*Give P values as exact values whenever suitable.*
☒	☐	For Bayesian analysis, information on the choice of priors and Markov chain Monte Carlo settings
☒	☐	For hierarchical and complex designs, identification of the appropriate level for tests and full reporting of outcomes
☐	☒	Estimates of effect sizes (e.g. Cohen's d, Pearson's r), indicating how they were calculated

Our web collection on statistics for biologists contains articles on many of the points above.

Software and code

Policy information about availability of computer code

Data collection	All cell culture microscopy data was collected using a custom-built confocal laser scanning (inverted) microscope based on a Zeiss Axio Observer Z1 controlled by custom software and includes provisions for automated, multisite time-lapse microscopy. The software was written in C and C++ and is available upon request.<br>In vivo data was collected using a Prairie Technologies Ultima IV two-photon microscope, controlled by the company's software. Images of histological sections was collected using a Stellaris 8 FALCON confocal microscope, controlled by the company's software.
Data analysis	All cell culture imaging data analysis was performed using custom written software ('OpenView') which includes features for automated/manual tracking of individual objects and measurements of fluorescent intensities over time (described in detail in Kaufman et al., 2012). The software was written in C and C++ and is available upon request. Whenever provided, the corresponding author provides a personal tutorial in (done many times in the past)<br>In vivo data was analyzed using custom MATLAB processing pipelines (Muller thesis 2023; Eppler, 2022) described in detail in the text and in Supplementary Figure 10. All Matlab code is available upon request

For manuscripts utilizing custom algorithms or software that are central to the research but not yet described in published literature, software must be made available to editors and reviewers. We strongly encourage code deposition in a community repository (e.g. GitHub). See the Nature Portfolio guidelines for submitting code & software for further information.

Data

Policy information about availability of data

All manuscripts must include a data availability statement. This statement should provide the following information, where applicable:

- Accession codes, unique identifiers, or web links for publicly available datasets
- A description of any restrictions on data availability
- For clinical datasets or third party data, please ensure that the statement adheres to our policy

No large datasets were generated. However, new expression vectors were created. Their full sequences are available as supplementary information .

Research involving human participants, their data, or biological material

Policy information about studies with human participants or human data. See also policy information about sex, gender (identity/presentation), and sexual orientation and race, ethnicity and racism.

Reporting on sex and gender	N/A
Reporting on race, ethnicity, or other socially relevant groupings	N/A
Population characteristics	N/A
Recruitment	N/A
Ethics oversight	N/A

Note that full information on the approval of the study protocol must also be provided in the manuscript.

Field-specific reporting

Please select the one below that is the best fit for your research. If you are not sure, read the appropriate sections before making your selection.

☒ Life sciences ☐ Behavioural & social sciences ☐ Ecological, evolutionary & environmental sciences

For a reference copy of the document with all sections, see nature.com/documents/nr-reporting-summary-flat.pdf

Life sciences study design

All studies must disclose on these points even when the disclosure is negative.

Sample size	All cell culture data were obtained from multiple separate experiments (typically 3 or more) from separate cell culture preparations made from separate rat pup litters. Data collected from tens of synapses per neuron, from multiple neurons per experiment. Data from individual neurons shown separately in almost all figures.
Data exclusions	Experiments in which technical failures occurred (e.g. perfusion, heating, CO2 supply, power outages, contamination) were excluded. Otherwise, all data were included.
Replication	All cell culture data were obtained from multiple separate experiments (typically 3 or more) from separate cell culture preparations made from separate rat pup litters. Data collected from tens of synapses per neuron from multiple neurons per experiment. All information on numbers of experiments, neurons, and synapses are provided explicitly in figures, figure legends and main text
Randomization	No explicit randomization was carried out. Some of the experiments were carried out sequentially, but many were interleaved with other experiments.
Blinding	No explicit blinding was performed

Reporting for specific materials, systems and methods

We require information from authors about some types of materials, experimental systems and methods used in many studies. Here, indicate whether each material, system or method listed is relevant to your study. If you are not sure if a list item applies to your research, read the appropriate section before selecting a response.

Materials & experimental systems

n/a	Involved in the study	
☐	☒	Antibodies
☒	☐	Eukaryotic cell lines
☒	☐	Palaeontology and archaeology
☐	☒	Animals and other organisms
☒	☐	Clinical data
☒	☐	Dual use research of concern
☒	☐	Plants

Methods

n/a	Involved in the study	
☒	☐	ChIP-seq
☒	☐	Flow cytometry
☒	☐	MRI-based neuroimaging

Antibodies

Antibodies used	FluoTag®-X2 anti-PSD95 Alexa 647 (NanoTag Biotechnologies #N3702 AF647-L; 1:200)<br>Mouse anti gephyrin primary antibody (Synaptic Systems #147 111; 1:500)<br>Mouse anti gephyrin primary antibody (Synaptic Systems #147 011; 1:1000)<br>Cy™5 AffiniPure conjugated polyclonal Donkey Anti-Mouse IgG (Jackson ImmunoResearch Laboratories, #715-225-150; 1:200)
Validation	Antibodies are broadly used commercial ones. #147 111 and #147 011 validated using KO animals by vendor. Partial validation carried out in house for Abd #147 011 (Supplementary Fig. 2)

Animals and other research organisms

Policy information about studies involving animals; ARRIVE guidelines recommended for reporting animal research, and Sex and Gender in Research

Laboratory animals	Wistar rats, newborn<br>Wild type C57BL/6J mice
Wild animals	N/A
Reporting on sex	No preference for either sex
Field-collected samples	N/A
Ethics oversight	Protocol for newborn rats approved by the Technion Israel Institute of Technology's Committee for the Supervision of Animal Experiments (approval IL-105-08-20).<br>Experiments with mice performed in accordance with the German laboratory animal law guidelines for animal research and approved by the Landesuntersuchungsamt Rheinland Pfalz (Approvals # G 17-1-051 and G 22-1-091).

Note that full information on the approval of the study protocol must also be provided in the manuscript.

Plants

Seed stocks	N/A
Novel plant genotypes	N/A
Authentication	N/A
